# Metallic nanoparticles: from biosynthesis to biomedical applications, current scenarios and prospects

**DOI:** 10.1039/d4na01054j

**Published:** 2026-01-13

**Authors:** Rabih Ajib, Krishnamoorthy Shanmugaraj, Ram Manohar Yadav, Tania P. Brito, Dinesh Pratap Singh

**Affiliations:** a Physics Department, Faculty of Science, University of Santiago of Chile (USACH) Av. Víctor Jara 3493, Estacion Central 9170124 Santiago Chile singh.dinesh@usach.cl dineshpsingh@gmail.com; b West Physics 3825 Paces Walk SE # 250 Atlanta GA 30339 USA; c Departamento de Química, Facultad de Ciencias, Universidad de Tarapacá Avda. General Velásquez 1775 Arica Chile; d Department of Physics, University of Allahabad University Road, Old Katra Prayagraj (Allahabad) Uttar Pradesh-211002 India

## Abstract

Metallic nanoparticles (MNPs) have attracted significant interest among researchers since the previous century owing to their vast potential applications in emerging fields such as nanotechnology, nano-optics, nanoengineering, nanoenergy, and biomedicine. The rapidly increasing demand for various MNPs has driven researchers to develop facile, inexpensive, scalable, and sustainable synthesis methods to explore their properties and potential for future applications across different scientific and industrial sectors. Due to their intrinsic physicochemical properties, such as surface plasmon resonance, biocompatibility, and luminescence behavior, MNPs have found numerous biomedical applications. Currently, these materials are synthesized and functionalized with different chemical groups, allowing them to conjugate with ligands, antibodies, and drugs of interest. This enables a wide range of applications in biotechnology, targeted drug delivery, magnetic separation, gene and drug delivery vehicles, and importantly, the diagnosis, imaging, and treatment of cancers. Key factors such as size-dependent melting temperature, surface plasmon resonance-based luminescence, and biocompatibility make MNPs highly valuable in bio-industrial applications. Various imaging modalities such as CT, MRI, SERS, ultrasound (US), and other optical imaging techniques have been developed to aid in disease detection and monitoring at various stages. The development of new biomedical techniques and applications requires a comprehensive understanding of the interactions between MNPs and target cells. This review focuses on different types of metallic nanoparticles, their advanced synthesis strategies such as biogenic approaches in addition to conventional methods, and their up-to-date biomedical applications including early detection, diagnosis, imaging, efficient drug delivery, and cancer therapy. Moreover, their antimicrobial activities against harmful bacteria, viruses, and fungi are discussed in detail. In addition, these nanoparticles are highlighted as optical contrast agents for bioimaging techniques such as SERS, MRI, and computed tomography, as well as for use in biosensors to detect biological molecules. Furthermore, by taking advantages of intriguing properties of various metals, biogenically synthesized bimetallic, mixed metal oxides, bifunctional composites, and graphene-based metal composites, can enhance the performance and need to be explored in future for advanced bio medicinal applications.

## Abstract 2

Metallic nanoparticles (MNPs) have attracted significant interest among researchers since the previous century owing to their vast potential applications in emerging fields of nanotechnology, nano-optics, nanoengineering, nanoenergy, and biomedicine. Figure partially created using Google Gemini.

## Graphical abstract

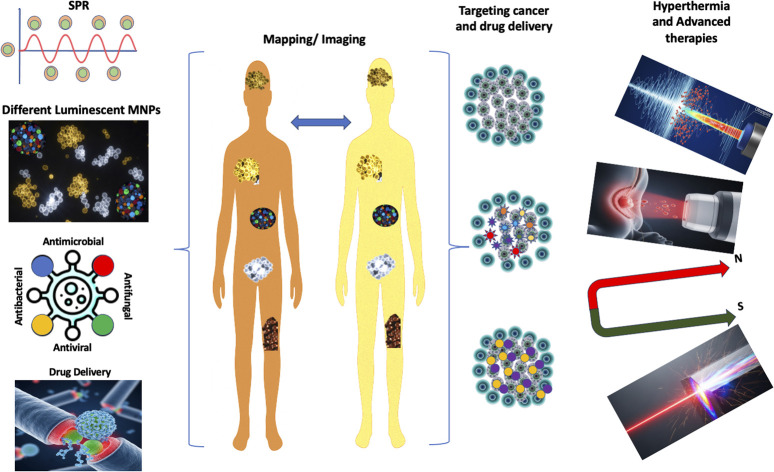

## 1. Introduction

Nanoscience and nanotechnology are the science and technology of nanomaterials, which involve the study of distinctive properties at the nanoscale by modifying the structures with complexes, composites and functionalizations and have a significant impact on several domains of investigation and industries.^1^ It is a considerably rapid growing field that studies small feature sizes with a broad range of applications. Nanotechnology is applied in many industries including electronics, textile fabrication, cosmetics, and construction.^1,2^

**Fig. 1:**
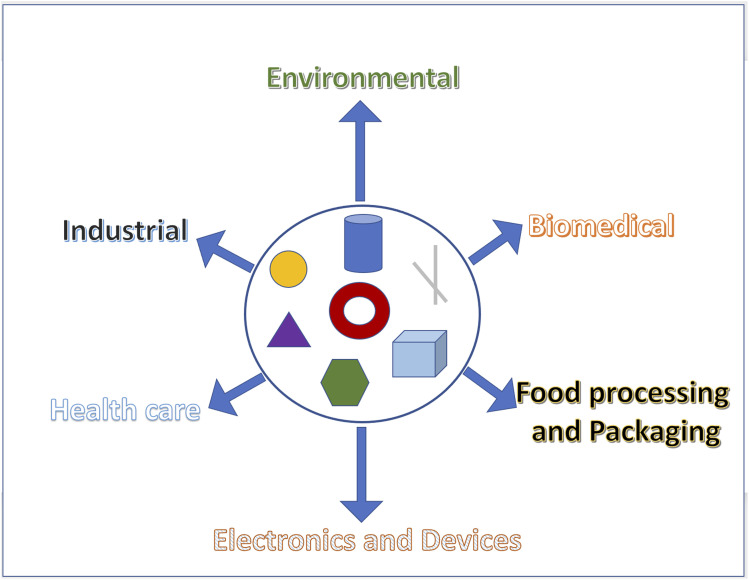
Various forms of metallic nanoparticles (such as nanoparticles, nanorods, nanotubes, nanohexagons, nanocubes, triangle and ring shaped) and their utilization in different research and industrial sectors.

Additionally, the automotive industries uses nanotechnology for fabricating structural and electronic materials such as fuel cells, lithium-ion batteries and supercapacitors.^3^ Nanomaterials are also applied for the delivery of chemicals^4^ or fluid filtration and water treatment.^5^ Besides the various applications of nanomaterials in biomedical sectors, they are widely used in medical research in numerous ways. For example, at present, various MNPs are utilized in drug carriers, drug delivery, cancer therapy, pharmaceutical applications, *etc.* Moreover, nanoparticles and quantum dots are used in the treatment of antibiotic-resistant bacteria. Nanoparticles, having a size range of 1–100 nm, exhibit a variety of phenomena that do not exist in their bulk counterparts.^5^ As a result of the decrease in size, the surface area increases exponentially, and thus the overall surface of a NP and its surroundings become more reactive.^6^ The chemical properties of NPs, including their chemical composition, surface chemistry, phase identity, and hydrophilicity, are also enhanced. However, the physical properties of NPs, including their shape, specific surface area, size and size distribution, aspect ratio, agglomeration, surface morphology, structure and solubility, play a major role in various applications. The magnetic properties of NPs originate from unequal electronic distribution. It has been determined that when the size of synthesized NPs is less than the critical value, magnetic properties can dominate, and depending on the choice of the applications, the mechanical properties of NPs must also be studied. In addition, NPs are expected to possess different thermal properties and behaviors, as most of them are usually synthesized in solvents such as oil, ethylene glycols or water to avoid agglomeration. It is noteworthy that the higher surface area of nanoparticles is beneficial for heat transfer along the NP surface. Recent research reports have mentioned that nanofluids containing copper oxide or aluminum oxide NPs in water or ethylene showed advanced thermal conductivity^5^ (I. Khan *et al.*, 2019). Among the characteristics of metallic NPs, physiochemical properties, size, and shape allow their application in biomedical and environmental fields, such as catalysis, imaging, water treatment, storage, energy production, and drug delivery.^7,8^Fig. 1 summarizes the main applications of nanoparticles, specifically metallic nanoparticles (MNPs) that demonstrate strong UV-Vis spectra and show size- and shape-dependent optical properties. The biomedical applications of MNPs are attributed to their intrinsic physiochemical properties: as the size decreases, the high surface area of the particles gives rise to distinctive behaviors.^9^ There are several bottom-up or top-down approaches for the synthesis of MNPs, which result in different properties such as morphology, uniformity, shape, and size, allowing the detailed study of the novel properties of NPs.^9^ However, challanges in terms of controlled growth of uniform shape/size NPs, large scale production, facile and ecofriendly approaches for the synthesis, always restrict the realization of potential applications of NPs. There are various reviews available on the synthesis of nanomaterials; therefore, we are not focusing on that. The present review discusses the latest applications of MNPs, in particular their biomedical applications in different sectors.

## 2. Metallic nanoparticles (MNPs)

MNPs are composed of inorganic metal or metal oxides that are usually covered with an organic or an inorganic material shell or a shell that is also made of metal oxides. In other words, the nanoparticles that are synthesized from metallic precursors are called MNPs.^5^ MNPs possess lots of features that make them applicable in various domains. One of the most widely studied metallic NPs are gold nanoparticles (Au-NPs) because of their great potential in biomedical applications. Gold is recognized as a quintessential noble metal, and its NPs present unique properties such as ease of functionalization, catalytic activity, non-toxicity, and physicochemical and biological properties. Due to these characteristics, Au-NPs have various applications like biomedical imaging, analytical science, sensing, therapeutics, catalysis, medical diagnosis, and drug delivery.^7–13^ Au-NPs are also derived in colloidal form, that is, a colloid of gold particles of nanometer size. These interesting optical properties of the Au-NPs are attributed to their unique interaction with light.^14^

Due to their unique optical properties, Au-NPs have gained considerable research interest, with vast applications in fields including biological imaging, electronics, and materials science.^15^ Au-NPs can also be used for the delivery of therapeutic agents, which can be easily coated onto the surface of Au-NPs.^16^ This is due to the large surface area-to-volume ratio of gold nanoparticles that allow their surface to be coated with hundreds of molecules (including targeting agents, therapeutic drugs, and anti-fouling polymers). Au-NPs are also used in medical diagnostics. They are efficient detection biomarkers used in the diagnosis of cancers, infectious agents, and heart diseases.^17^

Another type of MNPs that can be applied in the biomedical domains are silver nanoparticles (Ag-NPs). Ag-NPs have unique physical, chemical, and optical properties that are being implemented for a wide variety of applications. The optical properties of Ag-NPs are of great interest due to the strong coupling to specific wavelengths of incident light. This gives them a tunable optical response and can be employed to build ultra-bright reporter molecules, highly efficient thermal absorbers, and nanoscale “antennas” that amplify the strength of the local electromagnetic field to detect changes in the nanoparticle environment. Both Au- and Ag-NPs have shape-dependent optical properties.^18^

Specific properties of Ag-NPs such as quantum size, surface, quantum tunneling, and volume effects enable them to serve as catalysts, chemical probes, antimicrobial materials, anti-cancer materials, chemical sensors, and drug delivery carriers. These properties depend on the particle distribution, size, and shape of the Ag-NPs.^18^ Like Au-NPs, ionic silver is also considered as a potential candidate for biomedical applications. Currently, Ag-NPs are widely integrated into a wide range of medical devices, including bone cement, surgical instruments, and surgical masks. In addition, it has been reported that Ag-NPs, in right quantities, are suitable for wound healing.^19–21^ In short, MNPs are currently applied to aid various disease states, but the advances in biomedical imaging depend largely on the shape, size, and selectivity of the nanoparticle to the target.^22^ Ag-NPs exhibit a large spectrum of bactericidal and fungicidal activities. Besides, Ag-NPs present a wide range of applications such as anti-oxidative, antiviral, anti-bactericidal, anti-tumor, anti-inflammatory, and antiangiogenic properties, and diagnosis of cancer/HIV/AIDS along with biological sensing, imaging, drug carriers, and chemical sensing. The nanotechnology of silver is a rapidly growing technology in the domain of orthopedics because of its antimicrobial properties. Moreover, Ag-NPs can be utilized in various applications such as tumor prostheses, trauma implants, bone cement, and hydroxyapatite coating to inhibit the formation of biofilms. The encouraging and favorable results of *in vitro* and *in vivo* studies of the use of Ag-NPs in this domain reduce the risk of infection in an efficient and biocompatible manner. In addition, Ag-NPs can be deployed in pharmaceutical and cosmetic fields as they are not dangerous to pigments and human health but may have health benefits.^23^

**Fig. 2:**
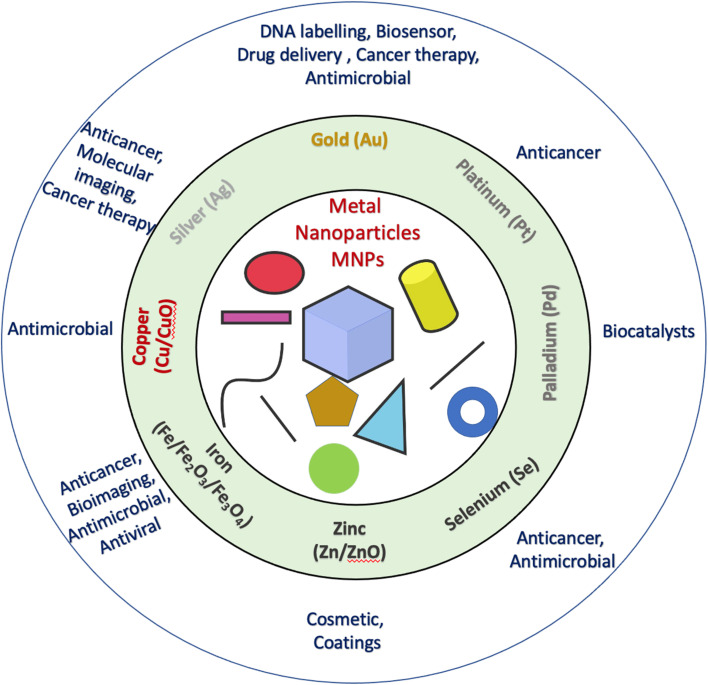
Different types of MNPs with various forms and their specific biomedical applications in relevant areas of research and investigations.

This review focuses on the recent advances in biomedical applications of MNPs. Fig. 2 gives a well-explained summary of the different types of MNPs and their specific biomedical applications. Among various MNPs, Ag and Au have a profound impact and a great potential in present research scenarios. However, the hope and futuristic applications of other nanoparticles also cannot be ignored.

## 3. Synthesis strategies of MNPs

**Fig. 3:**
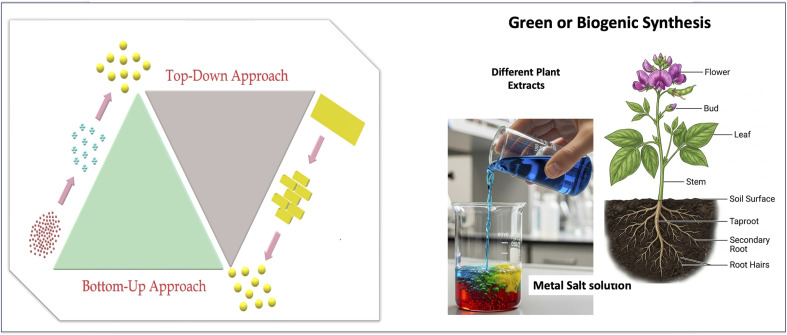
Schematic of the synthesis of MNPs by top-down (from bulk to nano sizes), bottom-up (from atoms and molecules to nano sizes) and GBMNP approaches by utilizing metal salts and different plant extracts. Figure partially created using Google Gemini.

These MNPs are facile to synthesize, although high-purity homogeneity is still a challenge. Usually, MNPs can be synthesized by two well-known approaches, called bottom-up and top-down approaches (left part, Fig. 3).^24^ The bottom-up approach refers to the build-up of material from the bottom: atom by atom, molecule by molecule, or cluster by cluster. In contrast, top-down approach refers to the synthesis of NPs from bulk materials, reduced to nano-dimension by utilizing various chemical, physical, and biological methodologies. Top-down approaches widely use externally controlled equipment such as milling, cutting, and shaping the materials into the desired shape and order.^25^ Various physical techniques such as lithography,^26^ photo/e-beam lithography,^27^ spray pyrolysis,^28–30^ ultrasonic spray pyrolysis,^31^ and radiation-induced^32,33^ methods belong to this group. A main drawback of this approach is the production of imperfect shape of metal NPs, which significantly affects their chemical and physical properties. Recently, some other new techniques have been used for the synthesis of MNPs by top-down approaches such as soft, scanning, colloidal, nanoimprint, and E-beam lithography.^34,35^ Theoretically, all these methods use either electrons, light, electrostatic forces, or a focused beam of electrons to selectively remove nanomaterials from their precursor to develop ordered arrays of MNPs. In addition, pyrolysis is another frequent method used for the synthesis of MNPs. Here, the metal precursors in their vapor state are forced through an orifice with high burning and MNPs are recovered from its solid ash. In addition, a lot of energy is necessary to maintain high-temperature and high-pressure conditions during these synthetic protocols, which makes these protocols costlier.

However, the bottom-up strategy for the synthesis of MNPs originates from individual molecules because it involves biological or chemical reduction. Usually, the chemical reduction method involves two important stages that are nucleation and successive growth. When the growth and nucleation are completed in the same stage, it is called *in situ* synthesis; or else it is called seed-growth method.^36,37^ The principle of the chemical reduction method is the reduction of metal precursors with a suitable reducing agent with a stabilizing agent to improve its stability. There are lots of reducing agents used in this procedure such as hydrazine, NaBH_4_, sodium citrate, LiAlH_4_, alcohols, and hydrogen.^38^

The shape, size and stability of the synthesized metallic nanoparticles are dependent on various factors such as reducing agents, pH, reaction time, temperature and metal ion concentration. Further, the pH of the medium plays an important role in modulating the shape and size of the MNPs. Controlling the pH of a solution, specifically by adjusting the concentration of OH^−^ ions, directly influences the size and shape of gold nanoparticles.^39^ An increase in pH was found to produce smaller, more uniform spherical nanoparticles with a tighter size distribution. Moreover, increasing the temperature can speed up the synthesis process, resulting in slightly larger nanoparticles. Therefore, both pH and temperature are crucial factors that can be manipulated to optimize the synthesis of gold nanoparticles. Higher alkaline conditions tend to produce small size and relatively stable Ag nanoparticles. The shapes of the nanoparticles, such as circular, triangular, and hexagonal, were controlled by varying the pH to 4, 5 and 11, respectively.^40^

Another important method for the synthesis of MNPs is the laser ablation process. Here, the solid surface is irradiated with a laser beam, and the materials become heated at low laser flux and are finally evaporated or sublimated.^41^ Microwave-based synthesis is also used to synthesize MNPs from their salts and polymeric surfactant solutions.^42,43^ Several other methods are also utilized for the synthesis of MNPs such as biogenic, electrochemical,^44^ thermal decomposition,^45^ and tip-directed synthesis.^46^Fig. 3 shows the main methods used to synthesize MNPs.

## 4. Green or biogenic synthesis of MNPs

Green or biogenic metallic nanoparticle's (GBMNPs) synthesis approaches involve an ecofriendly and sustainable way to create nanoparticles. Although GBMNP synthesis is a kind of bottom-up approach, unlike conventional physical and chemical methods that often involve toxic chemicals, consume more energy, and generate hazardous by-products, green synthesis utilizes biological entities or natural products to reduce metal ions and stabilize the resulting nanoparticles. GBMNPs, synthesized through biological processes, are gaining significant attention because of their small size and high surface area, offering advantages over chemically produced nanoparticles due to their eco-friendly, sustainable, and biocompatible nature. The superior cytocompatibility and stability of BMNPs are due to their capping layer, and reduce toxicity and side effects, which make them a safer option for various biomedical applications. Researchers have extensively explored GBMNPs for their antibacterial activity, ability to sense heavy metals, and roles in food preservation, while also investigating their therapeutic potential in drug delivery, imaging, fighting nosocomial pathogens, and targeting cancer cells. The various characterization techniques for GBMNPs and their cost-effective, eco-friendly biogenic synthesis using agricultural and biological materials, emphasizing their diverse applications in medicine, analytics, food preservation, and consumer goods, along with their toxicological considerations are well discussed.^47^ This review explores the synthesis of MNPs *via* green chemistry, focusing on their application as novel drug delivery systems in anticancer and antimicrobial treatments. It surveys the production, characterization, physicochemical properties, and biological activities of these green-synthesized NPs. Kazemi, S. *et al.*^48^ extensively analyzed recent studies employing the *in silico* design for green synthesis and computational modeling to understand NP-target interactions. The proposed computational approach not only elucidated biological mechanisms but also predicted potential bioactivities, offering new perspectives for the future of green chemistry in smart medicine and modern disease therapies.

### 4.1 Biosynthesis of Au-NPs

Among all MNPs the, Au-NPs have shown high performances and particular promise for cancer diagnosis, precise drug delivery, and site-specific targeting, which has opened a new avenue in oncology. The advantages of biogenetically generated Au-NPs over other MNPs in cancer therapy, emphasizing novel “greener” synthesis strategies while highlighting the drawbacks of conventional NP preparation methods, have been described by T. Bhattacharya *et al.*^49^ The quasi-spherical Au-NPs with a mean diameter of 20–50 nm was reported by using a green plant extract of *Citrus aurantium*. These biologically synthesized Au-NPs demonstrated significant *in vitro* cytotoxic and anti-gastric carcinoma effects against several cancer cell lines in a time- and concentration-dependent manner.^50^

An extracellular extract from the fungus *Schizophyllum commune* was utilized to synthesize spherical Au-NPs with an average size of 90 nm, which exhibited significant antifungal activities against *Trichoderma* sp. and *Aspergillus flavus*. Furthermore, the Au-NPs demonstrated dose-dependent cytotoxic effects on A549 lung cancer cells, increasing the intracellular reactive oxygen species (ROS) which could be a potential candidate as a multi-functional therapeutic agent.^51^

An ecofriendly, one-pot method was adopted for the synthesis of Au-NPs loaded in saponin niosomes using *Sapindus mukorossi* pericarp extract as a reducing and stabilizing agent. The synthesized Au-NPs demonstrated significant antibacterial activity against *Staphylococcus aureus* at low concentrations. Furthermore, these nanoparticles exhibited anti-inflammatory potential and a dose-dependent anticancer effect across various human cancer cell lines, with notable cytotoxicity against MCF-7 cells.^52^

### 4.2 Biosynthesis of Ag-NPs

Biological synthesis methods address the limitations of conventional methods, providing a sustainable way to produce Ag-NPs for diverse uses. Ultimately, the versatility of green-synthesized Ag-NPs positions green nanotechnology as a promising future for medicine, enabling the creation of low- or non-toxic nanomaterials for antimicrobial, anticancer, anti-inflammatory, and drug-delivery applications.^53^ Biologically synthesized Ag-NPs, particularly those utilizing plant extracts, offer a safer, more economic, and environmentally friendly alternative, yielding biocompatible Ag-NPs with enhanced biological properties. These kinds of nanoparticles are highly useful for biomedical applications due to their excellent antimicrobial properties, but traditional chemical and physical synthesis methods raise concerns about toxicity, high cost, and energy consumption.

Among noble metals, Ag-NPs are also explored tremendously from the point of view of synthesis and biological applications. A sustainable synthesis of Ag-NPs by using extracts from *Randia aculeata* cell suspension cultures was reported, which demonstrated that higher pH levels lead to a greater concentration of polydisperse nanoparticles ranging from 10 to 90 nm. Moreover, these green-synthesized Ag-NPs exhibited significant antibacterial activity against several pathogenic bacteria and a strong antiproliferative effect on cancer cells, particularly at pH 6.^54^

A microwave-assisted green method was adopted for the synthesis of Ag-NPs with *Macrolepiota procera* mushroom extract, and these nanoparticles exhibited significant anticancer activity against various human cancer cell lines by inhibiting heat shock proteins (HSPs) and inducing apoptosis.^55^

The significant role of Ag-NPs in nanobiotechnology and medicine, primarily due to their potent antibacterial properties and diverse applications, is described by A. Kaushal *et al.*^56^ The structural aspects of Ag-NPs, including size, shape, and surface area, are crucial for tailoring their efficacy in various fields, from medicinal uses to agricultural productivity. Moreover, different methods for producing less harmful Ag-NPs with controlled sizes and forms were described. In addition, the generation and mechanisms of Ag-NPs, along with their anticancer, anti-inflammatory, antibacterial, antiviral, and anti-angiogenic properties were discussed.

Particularly, Ag-NPs and Au-NPs are reviewed from various points of view such as properties, synthetic and characterization methods, roles in cellular targeting, imaging, drug delivery, and cancer theranostics.^57^ The extract from therapeutically valuable plants of the Moraceae family has extensively explored up to decades for the sustainable synthesis of MNPs like gold, silver, copper, and palladium. These plant extracts, rich in compounds such as flavonoids and terpenes, have been used to create nanoparticles that are then analyzed with advanced methods and evaluated for applications in dentistry, combating antimicrobial resistance and cancer prevention.^58^

Similarly, researchers explored the biogenic synthesis of MNPs by using plant extracts, specifically preparing green tea extracts with three distinct deep eutectic solvents instead of water. These different solvents based on green tea extracts significantly boosted the biosynthesis of Ag-NPs, primarily because they contained up to 235% more catechins, which act as natural reducing and capping agents, leading to higher synthesis efficiency. Notably, extracts prepared with specific solvents such as glycerol and betaine with a common urea molecule yielded Ag-NPs with superior properties, including uniformly smaller sizes (39.12 ± 5.33 nm and 43.11 ± 6.42 nm, respectively) and improved dispersion. Moreover, these specific solvent-based Ag-NPs exhibited promising *in vitro* anti-cancer activity, comparable to doxorubicin, which also suggested that deep eutectic solvents can serve as effective extraction solvents and surface modifiers for enhanced nanoparticle applications.^59^

Besides plant extracts, other natural sources like bacteria, fungi, and biopolymers are now being utilized as effective capping and reducing agents for synthesizing Ag-NPs. The properties and applications of these Ag-NPs are heavily influenced by their synthesis conditions and their diverse and effective applications in fields like biosensors, MRI, cancer treatment, and antimicrobial agents.^60^

Researchers have efficiently employed shallot (*Allium cepa var. aggregatum*) extract in a one-pot green production method to synthesize Ag-NPs, leveraging the plant's chemical content to both reduce metal ions and stabilize the resulting nanoparticles. The green synthesized, spherical, well-dispersed metallic Ag-NPs with a size range of 35 ± 8 nm exhibited a significant antioxidant activity against the DPPH substrate and demonstrated effective antibacterial activities against both Gram-negative (*Escherichia coli*) and Gram-positive (*Staphylococcus aureus*) organisms. Furthermore, it showed promising cytotoxicity against the MCF-7 human breast cancer cell line.^61^

### 4.3 Biosynthesis of other nanoparticles

Plant phytochemicals have emerged as a unique and eco-friendly method for synthesizing MNPs, owing to their dual role as both capping and reducing agents. Besides silver and gold nanoparticles, other metallic nanoparticles are being explored for their biomedical applications. For example a green synthesis method was adopted for copper nanoparticles (Cu-NPs) using *Phragmanthera austroarabica* extract and optimization of conditions produced spherical, crystalline Cu-NPs with an ideal size of 44.6 ± 2.7 nm. These biosynthesized Cu-NPs demonstrated remarkable efficiency as probes for detecting hexavalent chromium ions with an exceptionally low detection limit of 1.20 nM and also showed excellent degradation capabilities for industrial organic dyes like Congo red and methylene blue. Furthermore, it exhibited high antioxidant activity, significant inhibition of human breast cancer cells (MDA-MB-231), and potent antibacterial and antifungal effects.^62^

The green synthesis of Cu-NPs by using *Piper nigrum* fruit extract was also reported by M. Kiranmayee *et al.*^63^ These Cu-NPs demonstrated significant therapeutic potential exhibiting potent antioxidant, antibacterial, and cytotoxic activities against *Staphylococcus aureus*, *Escherichia coli*, and MCF-7 breast cancer cells. Furthermore, *in silico* molecular docking identified key *Piper nigrum* compounds with promising druggability against relevant biological targets.

Utilizing a Phyto reducing approach, iron nanoparticles (Fe-NPs) with diameters ranging from 45 to 100 nm were successfully synthesized using an aqueous extract of *Vitex leucoxylon* leaves, obtained *via* a cold extraction method by Nahari *et al.*^64^ The formation of Fe-NPs was confirmed by a color change to black and characterization through different techniques, identified the role of carboxylic acids, unsaturated aldehydes, and ketones in the synthesis process as obtained in the plant extracts. Notably, these green-synthesized Fe-NPs exhibited significant antioxidant, anti-inflammatory, cytotoxic, and wound-healing activities *in vitro*, suggesting their potential as a dermal wound-healing agent and a cytotoxic agent against cancer.

Green methodologies for producing selenium nanoparticles (Se-NPs) employ non-toxic substances like plant extracts and microbial metabolites, making the process both practical and cost-effective. These Se-NPs exhibit desirable characteristics for clinical applications, including high biocompatibility and a variety of beneficial activities such as antimicrobial and anti-cancer properties by Martínez-Esquivias *et al.*^65^

Monometallic and bi-metallic NPs synthesized from lemongrass and lemon, emphasizing their roles in healthcare and medicinal applications, were also reviewed. These plant-derived nanoparticles offer unique physical and chemical characteristics due to their size and shape. Overall, the lemongrass and lemon-derived nanoparticles demonstrated promising applications across various domains, showcasing their potential for future advancements (Farooq & Ngaini *et al.*).^66^

A summary of the different approaches and the basic descriptions are given in Table 1.

**Table 1:** Different synthesis strategies for MNPs, categorized by approach

Approach	Methods	Description
Top-down^25–35^	Mechanical milling (ball milling)	Grinding, milling of bulk materials for certain hours to fabricate nanocrystalline materials by means of attritor, Spex shaker or planetary ball mill
	Laser ablation	Using a laser to vaporize and condense nanomaterial onto different substrates
	Sputtering	Bombarding a target material with ions or evaporation of target materials to deposit a thin film of nanoparticles on some substrates
Bottom-up^36–46^	Chemical reduction	Using suitable metal salts and reducing agent to convert metal ions into MNPs
	Sol–gel	Forming a sol (colloid solution) and then a gel and finally resulting into nanoparticles
	Hydrothermal/solvothermal	By utilizing high-temperature stainless steel autoclaves and furnaces to react the reactants, soluble in water or some solvents to obtain synthesize nanoparticles/nanomaterials
	Chemical vapor deposition (CVD)	Depositing a thin film of nanoparticles onto a substrate from a gaseous phase and high temperature decomposition of reactants
	Physical vapor deposition (PVD)	Depositing a thin film of nanoparticles onto a substrate from a high vacuum-based vapor phase or inert gas condensation
Green synthesis^47–66^	Mostly by chemical/hydrothermal reduction of metal salts but by ecofriendly plants extracts or bacteria	Using biological agents (plants, bacteria, fungi) to synthesize nanomaterials. These extracts work as a reducing as well as stabilizing agent for the growth of MNPs

## 5. Biomedical applications of MNPs

**Fig. 4:**
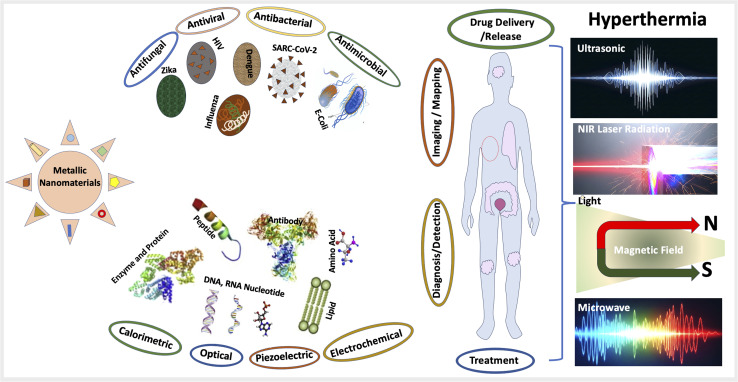
A representative image of the different types of MNPs and their biomedical applications. Representative images as antibacterial, antifungal, antiviral and antimicrobial agents and various types of biosensors to detect biomolecules. Use of nanoparticles in different stages of the cancer from diagnosis to imaging to drug delivery and treatment of the cancer by various approaches of hyperthermia techniques. *E. coli* images courtesy of National Human Genome Research Institute. Biological molecules schematic adapted with permission from ref. 223. Figure partially created using Google Gemini.

MNPs have emerged as an efficient tool for widespread biomedical applications. Fig. 4 summarizes the various biomedical applications of MNPs such as (i) anti-bacterial, anti-viral and anti-fungal agents, (ii) biosensors and (iii) cancer therapy including detection, mapping, and destruction of cancers cells.

### 5.1 As an anticancer agent

Cancer continues to be a global health crisis, and the harsh side effects of traditional treatments like chemotherapy and radiation highlight the urgent need for innovative, less toxic therapies. Nanotechnology offers a promising solution by enabling the eco-friendly production of anticancer nanoparticles, with metal-based nanoparticles being particularly noteworthy for their exceptional performance and utility in diagnosis, imaging and therapy. The widespread availability of plant-based biological precursors makes large-scale, ecofriendly synthesis of these metal nanoparticles feasible. The strong potential of phyto-based metal nanoparticles in combating cancer, exploring their mechanisms of action and emphasizing the benefits of green synthesis for efficient, non-toxic, and economical cancer therapy is described.^67^

Traditional cancer diagnosis has significantly improved with the use of NPs, which streamline the process and enhance efficiency. These nanoparticles, due to their unique properties and low toxicity to healthy cells, have garnered interest across various fields, making them valuable in the treatment and diagnosis of numerous diseases.^68^

The challenge of delivering chemotherapy effectively, minimizing side effects while maximizing efficacy, continues to drive research for new anticancer medications. Nanotechnology is revolutionizing medical research by offering innovative materials, particularly metal nanoparticles, that possess inherent or surface-induced anticancer properties, making them highly valuable as alternative therapeutic strategies. The rapid development of these metal nanoparticles is opening new avenues for improving cancer treatment outcomes, with a focus on targeted delivery and enhanced therapeutic effects.^69^

Various noble and noble metal-derived MNPs, such as Au, Ag, their oxides or alloys, have shown encouraging results in laboratory settings for treating a variety of cancers. Moreover, the tumor microenvironment influences the performance of MNPs, in terms of their associated toxicity.^70^

Studies on the cytotoxicity of biologically synthesized MNPs in various cancer cell lines were conducted by the facile synthesis of Au-NPs using phytochemicals present in grapes (*Vitis vinifera*) that act as reducing and stabilizing agents. The synthesized Au-NPs demonstrated a strong affinity towards human breast cancer cells (HBL-100) and induced apoptosis in exposed cells.^71^ Analysis and synthesis of medicinal plant *Indigofera tinctoria* leaf extract-induced biogenic Ag-NPs and Au-NPs are ongoing research.^72^ Silver and gold NPs demonstrated dose-dependent cytotoxicity to the lung cancer cell line A549, where NPs showed more toxic effects on cancer cell lines than the control (pure plant leaf extract). In another approach, cytotoxic properties of fungus (*Penicillium brevicompactum*)-mediated synthesized biogenic Au-NPs were analyzed in mouse myoblast cancer (C2C12) cells.^73^

The green synthesis of gold and Ag-NPs (Au-NPs/Ag-NPs) using diverse medicinal plants, a cost-effective and safe method that leverages the plants' inherent physical, chemical, and biological characteristics was reported by M. A. Raza *et al.*^74^ The significance of these biosynthesized Au-NPs/Ag-NPs as potent anticancer and antidiabetic agents was also highlighted.

The *Polygala elongata* leaf extract was utilized to synthesize 10–20 nm spherical Au-NPs, which acted as both a reducing and a stabilizing agent (Elangovan *et al.*, 2024).^75^ The synthesized Au-NPs demonstrated versatile applications, acting as an effective heterogeneous catalyst for methylene blue degradation under UV light, achieving 73% and 88% degradation rates. Furthermore, these biogenic Au-NPs exhibited strong antioxidant properties and a significant dose-dependent inhibitory effect on the A549 lung cancer cell line.

The green synthesis method for Ag-NPs has gained significant attention due to their biocompatibility, cost-effectiveness, and eco-friendliness, making them highly valuable in various biomedical applications, particularly in cancer diagnosis and therapy. Research showed that Ag-NPs exhibit anticancer activity through mechanisms like DNA damage, cell cycle arrest, and the induction of apoptosis.^76^

The cytotoxic and anticancer activities of phyto-reduced Ag-NPs and their molecular mechanisms in MCF-7 breast cancer cell lines were reported by I. Ullah *et al.*^77^ A green synthesis process, optimized using *Indigofera heterantha* aqueous leaf extract, yielded Ag-NPs approximately 12.7 nm in size, which were thoroughly characterized for their structural, vibrational, shape, and morphological properties using various spectroscopic and microscopic techniques. The Ag-NPs demonstrated a concentration-dependent inhibitory effect on MCF-7 cells with an IC50 of 27.93 ± 2.10 µg mL^−1^, which showed significantly higher selectivity towards cancer cells compared to L929 normal cells. Further investigation revealed that these Ag-NPs exert their cytotoxic and apoptotic effects on MCF-7 cells by damaging membrane integrity and inducing nuclear fragmentation, primarily due to oxidative stress generated by elevated levels of reactive oxygen species (ROS).

R. Salih *et al.*^78^ synthesized Ag-NPs capped with *Noctiluca scintillans* algae extract, identifying several phytochemicals with anticancer activity, such as *n*-hexadecanoic acid and beta-sitosterol, within their shell. When tested on MDA-MB-231 human breast cancer cells, these Algae-Ag-NPs demonstrated a time- and dose-dependent reduction in cell viability, notably inducing apoptosis specifically in cancer cells while having a less potent effect on normal cells. Furthermore, *in vivo* studies using a breast cancer xenograft model revealed a significant reduction in tumor growth in mice treated with Algae-Ag-NPs.

A simple, cost-effective, and eco-friendly biomimetic synthesis of spherical, non-aggregated Ag-NPs of size 20–70 nm utilizing the flavonoid extract of *Perilla frutescens* (PFFE) as a bio reduction agent was reported by T. Y. Hou *et al.*^79^ DLS studies further revealed a monodispersed nature with an average hydrodynamic size of 44 nm and a highly negative zeta potential, indicating excellent stability. These PFFE-Ag-NPs exhibited significant cytotoxic effects against human colon carcinoma (COLO205) and mouse melanoma (B16F10) cell lines, with IC50 concentrations of 59.57 and 69.33 µg mL^−1^, respectively. Moreover, PFFE-Ag-NPs demonstrated potent antibacterial activity against both Gram-positive and Gram-negative pathogens, along with considerable *in vitro* antioxidant activity by scavenging DPPH and H_2_O_2_ free radicals. These PFFE-Ag-NPs exhibited promising potential as a multifunctional nanomaterial for biomedical applications, particularly in cancer therapy and infection control.

The biosynthesis of Pt, Au, and Ag nanoparticles using a native bacterium (GFCr-4), along with Au–Ag alloy and Au@Ag core–shell bimetallic nanoparticles, was reported by H. Mollania *et al.*^80^ The synthesis conditions were optimized by varying effective parameters such as pH, temperature, and electron donors, which resulted uniformly distributed nanoparticles with average sizes ranging from 25 nm (Ag) to 86 nm (Au@Ag core–shell). The synthesized nanoparticles demonstrated excellent catalytic performances; for instance, Au–Ag alloy nanoparticles showed superior catalytic activity in the Gewald reaction compared to monometallic Au-NPs, and Pt-NPs boosted photocatalytic yield by up to 92% under green LED illumination. Furthermore, Pt-NPs exhibited low toxicity to normal 3T3 cells but significantly inhibited MCF-7 cancer cells by up to 77.3%, highlighting their potential for biomedical applications.

The potential of Cu nanoparticles as drug delivery agents, a less-explored area compared to Au- and Ag-NPs, was investigated by focusing on their size, surface control, and unique magnetic and near-infrared absorption properties. B. A. Akash *et al.*^81^ synthesized beta-cyclodextrin citrate-coated spherical Cu nanoparticles of size around 10 nm *via* a hydrothermal method. Importantly, these nanoparticles achieved high encapsulation efficiency (over 94%) of the chemotherapy drug 5-fluorouracil, with sustained release over 12 hours. *In vitro* cytotoxicity studies on breast cancer cells (MDA-MB-231) demonstrated dose-dependent effects for both empty and drug-loaded nanoparticles, confirming the suitability of beta-cyclodextrin citrate-coated Cu nanoparticles as effective nanocarriers for 5-fluorouracil.

In another approach, spherical Cu-NPs ranging from 38 nm to 94 nm were synthesized by utilizing Himalayan *Hippophae rhamnoides* L. stem extract.^82^ These synthesized Cu-NPs demonstrated significant anticancer activity against HeLa cancer cells, exhibiting a concentration-dependent reduction in cell viability with an IC50 value of 48 µg mL^−1^. Further investigation revealed that the Cu-NPs induced apoptosis in HeLa cells, evidenced by increased reactive oxygen species (ROS) production and notable nuclear condensation.

Furthermore, some other nanoparticles such as palladium(ii) and platinum(ii) including bimetallic forms were synthesized ecofriendly by utilizing extracts from rosemary and ginseng due to their known anticancer potential.^83^ Their efficacy as anticancer agents was evaluated *in vitro* against colon cancer cell lines (Ls180 and SW480). Findings revealed that these nanoparticles successfully induced cell death and demonstrated a more pronounced effect than the plant extracts alone. Furthermore, the study investigated the nanoparticles' influence on autophagy markers and apoptosis, indicating a significant impact on these cellular processes. Ultimately, this research bridges a gap in current literature by comprehensively exploring the anticancer properties of palladium and platinum nanoparticles biosynthesized from these specific medicinal plant extracts, highlighting their potential for future medical applications in cancer treatment.

### 5.2 MNPs in conventional cancer therapies

Cancer, marked by the uncontrolled proliferation of tumor cells, remains a major global cause of death for both men and women, with its development linked to carcinogens and even conventional therapies like radiotherapy and chemotherapy. MNPs can offer promising solutions for diagnosis and treatment due to their customizable properties and ability to target cancer cells specifically. They can be functionalized with various ligands for precise drug delivery, drug release, gene therapy, and photothermal therapy, making them versatile in cancer management.^84^

The use of NPs in traditional cancer diagnosis has revolutionized the process by enhancing efficiency and targeting capabilities while minimizing toxicity to healthy cells, making them valuable in the treatment and diagnosis of various diseases. The advancements in nanotechnology stem from the development of engineered MNPs, which have gained significant attention in the biomedical field due to their inert nature and nanoscale structures that resemble biological molecules. The properties of these nanoparticles and their diverse applications in cancer imaging and therapeutics, while also addressing the challenges related to their clinical translation, are described (Khursheed *et al.*, 2022).^85^ The use of MNPs in conventional cancer therapies are described below.

#### 5.2.1 Radiotherapy

Radiotherapy, also known as radiation therapy, is a method for cancer treatment by damaging the DNA inside cancer cells, preventing them from growing and dividing. This process uses high doses of radiation to kill cancer cells and shrink tumors. High doses of radiation are always a risk that can also affect healthy cells. Radiotherapy can be done by two ways: first by; (i) external beam radiation therapy (EBRT), where radiation machine from outside directs the beam from various angles to precisely target the tumor while sparing healthy tissue. Technologies like intensity-modulated radiation therapy (IMRT), stereotactic body radiation therapy (SBRT), and proton therapy are advanced forms of EBRT that offer even greater precision. Or second by (ii) internal radiation therapy (Brachytherapy), where a radioactive source is placed directly inside or very close to the tumor and then exposed to the radiation, so that a very high dose of radiation to the tumor can be delivered, with minimal exposure to nearby healthy tissues. Radiotherapy can be used to cure cancer either alone or in combination with other treatments like surgery or chemotherapy. Moreover, it can be used to shrink a tumor before surgery making it easier to remove or to destroy the remaining cancer cells after surgery to prevent recurrence. Depending on the area of the body being treated, the dose of radiation, and the individual's overall health, radiotherapy can lead to some common side effects including fatigue, redness, dryness or peeling of skin and localized issues, nausea and vomiting for abdominal radiation, hair loss for head radiation, although most of the side effects are temporary and improve after treatment ends. At present, the process of radiotherapy bas been improved with higher efficiency by utilizing nanoparticles. For example, Au nanoparticles and nanorods are used as radio sensitizing agents in cancer therapy. A. Taheri *et al.*^86^ compared the efficacies of spherical and rod-like Au nanoparticles. By using Monte Carlo simulations, researchers evaluated how the size, aspect ratio, and orientation of GNRs affect secondary electron emission and dose enhancement. The findings suggest that GNRs, particularly those with smaller surface-area-to-volume ratios and alignment with the photon beam, are promising for enhancing radiotherapy, especially when considering their role in multimodal cancer therapy alongside photothermal therapy.

#### 5.2.2 Drug delivery and chemotherapy

Chemotherapy is a type of cancer treatment that uses powerful drugs to kill fast-growing cells in the body or to treat some other conditions like bone marrow diseases and certain immune system disorders. These efficient drugs work by interfering with the growth and division of cells. Different drugs target various stages of the cell cycle, which is the process by which cells grow, copy their genetic material (DNA), and divide to form new cells. Chemotherapy can be used to destroy and prevent the reoccurrence of the cancer and can be given as adjuvant or neoadjuvant therapies, *i.e.* after other treatments (like surgery or radiation) to kill any remaining cancer cells or before other treatments to shrink a tumor, making surgery or radiation more feasible, respectively. In the chemotherapy, the delivery of specific and optimum amount of dose of the drugs is crucial and very important for the effective treatment. Heavy dose of the drugs always causes destruction of the healthy cells, severe side effects and sometimes fatality of the patient. MNPs are being utilized extensively to enhance the chemotherapy efficacy and in targeted drug delivery for improved bioavailability, reduced side toxicity, and enhanced patient outcomes. The crucial role of MNPs in drug delivery, highlighting their aspect ratio, unique size, shape, and surface properties that enhance drug efficacy and reduce side effects, is well described by G. A. Croitoru *et al.*^87^ MNPs, including Au, iron oxide, and Ag nanoparticles, show promise as carriers for treating various diseases like inflammation and cancer, by improving drug solubility, bioavailability, and targeted release. However, the successful integration of MNPs into medical procedures necessitates addressing challenges such as potential toxicity, long-term safety concerns, and the need for standardized production methods. To achieve efficient targeted drug delivery, Au-NPs are utilized as carriers for the anticancer drug doxorubicin (DOX), forming a DOX-Au-NPs nanocomposite. Characterization revealed that both the Au-NPs and the DOX-Au-NPs nanocomposite were spherical and very small, having diameters of approximately 10 ± 2 nm and 12 ± 2 nm, respectively. Notably, the DOX-Au-NP nanocomposite significantly enhanced cytotoxicity against the MCF7 breast cancer cell line, with a concentration of 20 µM causing a decrease in cell survival similarly to 80 µM of free DOX, suggesting its potential for more effective cancer treatment (Faid *et al.*, 2022).^88^ Ag-NPs synthesized by using an aqueous extract from *Catharanthus roseus* leaves, which demonstrated a remarkable ability to rapidly reduce silver ions, confirmed the formation of spherical, uniformly sized Ag-NPs with a face-centered cubic structure. Significantly, these biosynthesized Ag-NPs exhibited potent antiproliferative and cytotoxic effects on HeLa229 cervical cancer cells, showed higher selectivity towards cancer cells than normal cells, and demonstrated robust antimetastatic potential by inhibiting cancer cell migration, suggesting a promising addition to conventional chemotherapy.^89^

In addition, MNPs in particular, serve as crucial platforms in nanomedicine, enhancing drug solubility, improving pharmacokinetic properties, and enabling high-efficiency, non-toxic, and specific cancer therapy. The development of MNPs aims to enhance the therapeutic efficacy by achieving site specificity, preventing multidrug resistance, and effectively delivering large doses of anticancer drugs directly to the target site, thereby avoiding off-target toxicity. Inorganic and organic NPs are evaluated *in vitro* and *in vivo* for the skin cancer therapy benefitted in terms of electrostatic charge, biodegradability, biocompatibility, high drug payload, and low toxicity, which resulted in improved response due to the higher accumulation of drugs in tumor tissues and better drug-release process in the conjugation of electromagnetic waves, temperature, pH, and light (Marzi *et al.*, 2022).^90^

Moreover, beyond inorganic and MNPs, polymeric, liposomal, and nanocrystal-based nanomaterials are currently attracting significant attention for cancer therapy due to their specific advantages such as targeted delivery and improved drug stability. The ongoing evolution of nanotechnology, including emerging protein-based nanoparticles and micelles, promises significant breakthroughs in nanomedicine and cancer care.^91^

#### 5.2.3 Immunotherapy

The immune system surveys the body for foreign invaders such as bacteria and viruses and abnormal cells, including cancer cells, and after identification, it can eliminate them. However, cancer cells often develop ways to evade immune detection and destruction. Immunotherapy is a revolutionary type of cancer treatment that harnesses the power of the body's own immune system to fight cancer. Unlike chemotherapy, which directly attacks dividing cells rapidly, immunotherapy works by either stimulating the immune system's natural defenses or providing it with additional tools to recognize and destroy cancer cells. Immunotherapies can be done by various ways such as immune checkpoint inhibitors, (ii) T-cell transfer therapy (adoptive cell therapy), (iii) monoclonal antibodies (mAbs), and (iv) immune system modulators (cytokines and other agents), and their details are beyond the scope of this review.

Given the limitations of conventional cancer therapies such as surgery, chemotherapy, and radiotherapy, there is a critical need for more reliable, less toxic, cost-effective, and specific approaches like immunotherapy, particularly for challenging cases such as breast cancer with its developed anticancer resistance.^92^ The efficacy of MNP-based breast cancer immunotherapy, focusing on their potential to provoke trained immunity or adapt innate immunity, was reported by Q. Du *et al.*^93^ Leveraging MNPs as a burgeoning field, the goal was to potentiate an immune response or directly combat cancer, especially given the immunosuppressive nature of the tumor microenvironment and poor immune cell infiltration. Although data on trained immunity's role in breast cancer cell elimination are scarce, this research introduces the promising potential of leveraging this aspect of immunity adaptation using MNPs.

Hepatocellular carcinoma (HCC) continues to pose a significant challenge due to its high recurrence and metastasis rates, with current apoptosis-based metallo immunotherapies proving inadequate in generating a robust immune response. Researchers developed a smart responsive bimetallic nanovaccine designed to overcome these limitations by inducing immunogenic cell death (ICD) through pyroptosis and enhancing the cGAS-STING immune pathway. This innovative nanovaccine, containing synchronized Fe^3+^, sorafenib (SOR), and Mn^2+^, is released in the tumor microenvironment to synergistically induce pyroptosis and expose critical immunogenic factors like double-stranded DNA (dsDNA). The released dsDNA and Mn^2+^ then collaboratively activate the cGAS-STING pathway, promoting dendritic cell maturation and Type I interferon release, while Mn^2+^ also enables T1 MRI for visual monitoring (Du *et al.*, 2024).^93^

## 6. Metal nanoparticles in advanced cancer therapy

Cancer remains a major global cause of death, particularly in late-stage cases with metastases that are largely untreatable and often result in prolonged chronic conditions. Nanotechnology offers a promising solution to this challenge, not only by enhancing current cancer treatments but also by developing innovative therapies that reduce systemic toxicity and improve targeted action on tumors and metastatic cells.^94^ Among the most studied nanoparticles for cancer treatment are metallic gold and Ag-NPs, quantum dots, polymeric nanoparticles, carbon nanotubes, graphenes, and even nanobubbles, all employing diverse mechanisms to improve outcomes such as inducing reactive oxygen species or enhancing targeted delivery (Pavelić *et al.*)^95^

### 6.1 MNPs in cancer therapy

There are different stages of cancer therapies from early detection to imaging or mapping of cancerous cells in the body and further targeted drug delivery, and finally, hyperthermia or photothermal therapy for drug release and destruction of the cancer cells. Nanotechnology distinguishes cancer cells from normal cells by active and passive targeting, which is important in cancer treatment. Metallic NPs are applied in cancer diagnosis, treatment, and monitoring all in a single product to enhance patient compliance and lessen potential harmful effects. Gold and silver NPs are applied in cancer imaging, photodiode therapy, hyperthermia, and tissue targeting, and they assist clinicians in the primary diagnosis and treatment of various cancers. Enhanced permeability and retention (EPR) effect and increasing concentration of nanoparticles in the tumor are possible by passive targeting, and active targeting (Fatema S. *et al.*, 2019)^96^ involves selective molecular recognition of antigens, often proteins, that are expressed on the cancer cell surface to localize NPs to malignant cells or take advantage of biochemical properties linked with malignancy such as matrix metalloproteinase excretion. Nanoparticles containing fluorophores, due to their luminescence property, have more applications in the diagnosis of cancer. Nanoparticles are regarded as new adaptable agents in treating cancer. Among several applications, metal nanoparticles (composed of high-Z atoms) were used as selective tumor cell radio sensitizers.^97^ Metal nanoparticles therefore offer a potential increase in radiotherapy efficiency that is influenced by metal nanoparticles with the minimization of its side effects. Gold and silver exhibit strong absorption and plasmon resonance light scattering and thus they have wide-ranging applications in radiation therapy, cell imaging, protein interaction, and DNA hybridization detection. Their facile surface chemistry and their exceptional optical properties make MNPs widely applied in cancer therapy. In addition, the appropriate size scale of silver and Au-NPs draws the interest of its usage in the diagnosis of cancer and its treatment. Silver and Au-NPs are widely used as anti-tumor agents in diagnosing tumors as well as in therapy by conjugating them with specific ligands and biomarkers. They are controlled by intravascularly or intraoperatively (S. Sadhasivam *et al.*, 2010).^98^ In addition, silver-based nanostructured materials can be employed as bio-imaging labels for human lung cancer H1299 cells.^99^ Silver NPs not only cause apoptosis but also sensitize cancer cells, and programmed cell death is concentration-dependent under certain conditions.^100^ Multifunctional silver-embedded magnetic nanoparticles made up of a thick silica shell with silver having an average size of 16 nm possess strong surface-enhanced Raman scattering (SERS) signals and have magnetic properties that allow them to be used for the treatment of breast cancer and leukemia by targeting breast-cancer cells (SKBR3) and floating leukemia cells (SP2/O) (R. Vijayan *et al.*, 2018).^101^ Biologically synthesized silver NPs show evidence of significant toxicity in MCF7 (breast cancer cells) and T47D cancer cells (epithelial cells isolated from a pleural effusion obtained from a patient with infiltrating ductal carcinoma of the breast) by higher endocytic activity than the MCF10-A normal breast cell line (M. Yafout *et al.*, 2021a).^102^ Many herbal-sourced silver NPs are used against cancer cells. Silver nanoparticles produced using *Origanum vulgare* exhibit dose-dependent response for human lung cancer (J. Beik *et al.*, 2016).^103^

However, the less harmful nature of Au-NPs because of their biocompatibility, simple and direct synthesis, chemical stability, and non-interference with biomarkers make them promising for the diagnosis of cancer.^104^ Au-NPs have distinctly absorbed laser light and are nontoxic. They can easily conjugate with proteins and antibodies. In addition, they have tunable optical properties. For all these reasons, they are the most appropriate candidates for photothermal sensitizing among other nanomaterials. The work done by H. H. Chang *et al.*^105^ on gold nanorods allowed tuning of the absorption peak of these nanoparticles, which can also be tuned from 550 nm up to 1 µm just by altering the aspect ratio of the nanorods. Hence, for the rod-shaped Au-NPs with absorption in the IR region, which selectively accumulated in tumors, laser light irradiation (in the IR region) barely warms the surrounding tissue, but the nanorods convert light to heat, causing the death of malignant cells, and this is one of the biggest recent successful applications of Au-NPs in photothermal therapy. Recently, a new type of nanoparticle developed that is known as ‘‘nano stars”, which pile up in tumor cells and scatter light, thus tumors become easily seen using special cameras (M. Yafout *et al.*).^102^ The size of these structures is about 140 nm across and contains 8-point gold stars that are encircled by a layer of dye and encased in a sphere of silica and a polymer. These nano stars enable the accurate detection of macroscopic malignant lesions, as well as microscopic diseases, without the need for a targeting moiety, in genetically engineered mouse models of breast cancer, in one human sarcoma xenograft model, and sarcoma as well. The sensitivity of surface-enhanced resonance Raman scattering nano stars allows imaging of premalignant lesions of pancreatic and prostatic neoplasia. High sensitivity and broad applicability, linked with their inert gold–silica composition, make SERRS nano stars promising imaging agents for more precise cancer imaging and resection (M. Yafout *et al.*).^102^ Moreover, the plasmonic heating response of nano stars serves as a signature of nanoparticle incorporation in cells, bringing the goal of nanoparticle-mediated photothermal therapy a step nearer. Gold nano stars can be used for simultaneous photoacoustic imaging and photothermal therapy in living cancer cells (D. Astruc *et al.*, 2010).^106^

This section highlighted and described the great impact of MNPs on cancer therapy. Such processes allow the differentiation of cancer cells from normal cells by active and passive targeting, which is crucial in cancer treatment. Metal nanoparticles are applied in cancer diagnosis, treatment, and monitoring, all in a single product enhancing patient compliance and minimizing potential unfavorable effects. Noble MNPs (silver and gold NPs) have several applications in hyperthermia, photodiode therapy, cancer imaging, and tissue targeting, and they assist clinicians in early diagnosis and treatment of various cancers.

After detection and exact mapping of cancer cells, the destruction process in an efficient and effective way is of utmost important. An untargeted drug delivery or inefficient destruction can cause re origination and also destruction of healthy cells, which can also result in the death of patients.

### 6.2 MNPs in hyperthermia

**Fig. 5:**
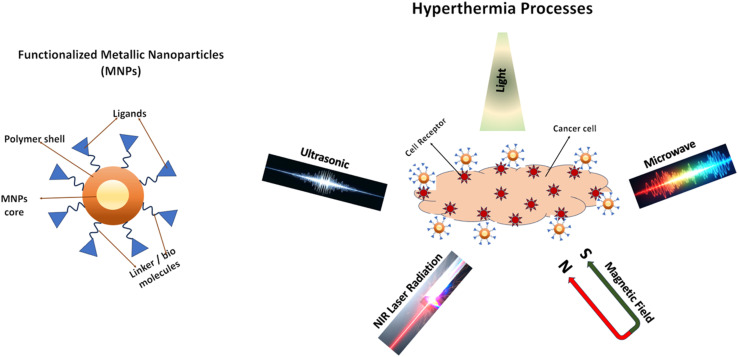
Schematic of the functionalized MNPs and their applications in cancer treatment *via* different approaches of hyperthermia treatment. Figure partially created using Google Gemini.

Hyperthermia, also known as thermal therapy or thermotherapy, is a broad term that refers to the medical treatment where body tissue is exposed to elevated temperatures, typically in the range of 40–45 °C. The heat can be applied locally, regionally, or to the entire body using various methods like microwaves, varying magnetic field, radio waves, ultrasound, or even heated fluids. The primary goal of hyperthermia is to damage and destroy cancer cells, which are often more sensitive to heat than normal cells. It is frequently used as an adjuvant therapy to enhance the effectiveness of other treatments like chemotherapy and radiotherapy, rather than as a standalone cure. Fig. 5 describes the functionalization of MNPs by different agents and their applications in cancer treatment *via* hyperthermia processes.

One of the medical concepts that have been used for a long time is heating process for therapeutic purposes. Commonly, heat was used for the ablation of cancer cells (like surgical removal of tumors) (H. Ueno *et al.*, 2018).^107^ It is important to define a way that uses lethal levels of heat to irreparably coagulate proteins and other biological molecules and induce cell death. This way is thermoablation (above 50 °C). A slight increase in temperature that does not cause cell death by itself may limit some of the collateral harm to normal tissues away from the ablation zone and could still have therapeutic benefits, which is defined as hyperthermia (with an assigned 41–50 °C temperature range). The usage of Au-NPs in hyperthermia is very beneficial because they are photothermally active and different shapes and sizes of Au-NPs have a range of melting temperatures. Therefore, modifying the size, shape, and composition of gold nanoparticles allows for the tuning of the resonance wavelength to the incident light.^108^ Hyperthermia is a treatment that requires applying heat to generate ablation of the tumor, improve drug delivery to tumors, improve tumor sensitivity to chemotherapy, or improve the immune response to cancer (A. Nakamura *et al.*).^109^ Gold is biologically compatible because it is an inert noble metal that is thermally and chemically stable, and it is also easy to conjugate biomolecules to its surface (for PEGylation or decoration with tumor-homing peptides and antibodies).

Another type of nanoparticles used in hyperthermia are core–shell Au-NPs. They have a layer of gold encircling a core made of dielectric material, most usually silica because it is biocompatible. This metal-dielectric structure triggers a redshift of the characteristic plasmon absorption spectrum of gold to the NIR region of the electromagnetic spectrum. The plasmon resonance frequency is tunable by shifting the core-to-shell ratio. GNSs measuring about 150 nm in diameter (120 nm silica core and 15 nm gold shell) are plasmon resonant at near-infra-red wavelengths and highly useful photothermal activators since their size allows for EPR-mediated passive accumulation in tumors, albeit not deep penetration into tumors. Covering their surface with covalent attachment of PEG that binds rigidly to the surface gold atoms renders them additional biocompatibility by minimizing opsonization, immune activation, and sequestration in the liver and spleen, thereby increasing their blood circulation time (J. Ando *et al.*, 2018).^108^

In preclinical toxicity, biocompatibility, and biodistribution studies accomplished with these GNSs before clinical use, there was no indication of toxicity for durations of up to 404 days (J. Ando *et al.*, 2020).^110^ Actively targeted Au-NPs to tumors in mice have demonstrated improvements in survival when compared to passively targeted nanoparticles (Liao *et al.*, 2019).^111^ Tumor photothermal ablation has progressed significantly in the past few years with multiple ongoing clinical trials based on an FDA IDE (investigation device exemption) granted for their use as AuroShell® particles (Nanospectra Biosciences, Inc., Houston, TX). Pilot studies were performed in prostate cancer patients to demonstrate passive accumulation in tumors upon intravenous administration (J. Ortega Arroyo *et al.*, 2014).^112^ Additionally, a favored sensitization of radioresistant cancer stem-like cells is being frequently studied. A study showed that breast cancer xenografts decreased in volume after radiation and even more so after radiation and GNS-mediated hyperthermia, but the relative proportion of stem-like cells increased in the radiated group, while they did not in the group treated with radiation and hyperthermia.^113^ Another MNPs that are currently used in hyperthermia are gold nanorods. GNRs are smaller (less than 50 nm typically). They have longer circulation times and are more efficient as photothermal activation agents (M. Liu *et al.*, 2017).^114^ The strong extinction coefficient of gold nanorods at the longitudinal surface plasmon resonance wavelength accounts for their potent photothermal activation properties, and this results in six-fold faster heating per gram of gold-nanofève than with gold nanorods (M. Shiota *et al.*, 2018).^115^ The tumor accumulation and anti-tumor efficacy of photothermal therapy following intravenous administration of GNRs are comparable to that following intra-tumoral administration of GNRs, as reported by J. Kneipp *et al.* (2006).^116^ Different approaches of hyperthermia are described in Fig. 5.

### 6.3 MNPs in photodynamic therapy (PDT)

Photodynamic therapy (PDT) is a two-stage medical treatment that selectively destroys abnormal cells, mostly used for certain cancers, precancerous conditions, and some skin and eye disorders. It is considered a localized treatment, as the light can only penetrate a limited depth of tissue, making it suitable for treating conditions on or just beneath the skin. In this therapy, first a specifically designed light-sensitive medicine, also called photosensitizer or photosensitizing agent, is introduced into the body by injecting it into the bloodstream, applying it as a cream to the skin, or by taking it orally. Second, after a specific time (hours to a few days), the affected area is exposed to a specific wavelength of light in the form of laser, LED, or even natural daylight for some skin conditions, so that the photosensitizer could be concentrated in the proximity of the target cells. This process is called light activation, and it activates the photosensitizer. Finally, when the affected area is exposed to light, the photosensitizer reacts with oxygen within the cancer cells to produce a highly reactive form of oxygen called singlet oxygen or other reactive oxygen species (ROS). These ROS are toxic to the cells, causing damage to cellular components and ultimately leading to the destruction of the abnormal cells. Besides this, PDT can damage the blood vessels, which supply oxygen and nutrients to the tumor, followed by starving and death, and PDT may even stimulate the body's immune system to attack the cancer cells, resulting in cell destruction.

MNPs can act as multifunctional nanoplatforms for targeted drug delivery and offer a distinct advantage over conventional drugs by enabling precise delivery of anticancer agents to specific tumor sites, thereby improving treatment outcomes and reducing complications. S. Shariatzadeh *et al.*^117^ specifically examined the application and impact of MNPs in modulating the tumor microenvironment, highlighting their potential in therapies such as photodynamic and sonodynamic therapies.

In the last decade, significant efforts have been devoted toward the synthesis of eco-friendly, green MNPs and their medical applications, as they offer advantages over traditional treatments and can be functionalized for targeted delivery. A. Chota *et al.*^118^ described the synthesis and therapeutic potential of these green-synthesized MNPs for enhanced cancer PDT. Moreover, the advantages of green hybridized activatable NPs over conventional photosensitizers were reviewed. Ultimately, the insights presented are expected to inspire the creation of new green nano-formulations for improved image-guided PDT in cancer treatment.

Biogenic Ag-NPs, synthesized using plant-mediated methods, are gaining significant interest in cancer research due to their cost-effectiveness, ease of production, and reduced toxicity compared to conventionally synthesized nanoparticles. These “green” Ag-NPs exhibited remarkable anticancer properties, which are attributed to their distinctive sizes, shapes, and optical characteristics. G. Kah *et al.*^119^ highlighted the recent progress in utilizing biogenic Ag-NPs as therapeutic agents for cancer treatment, focussed on their anticancer efficacy, underlying mechanisms, potential in PDT, utility in targeted cancer therapy, and overall toxicity.

### 6.4 MNPs in photothermal therapy (PTT)

PTT is an advanced medical treatment that utilizes light-absorbing materials that can generate heat after light irradiation and destroy targeted cells, most notably in cancer therapy. In the metal nanoparticles at room temperature there are positive metal ion cores surrounded by pool of negative electron cloud at the surface, which is called surface plasma. When light interacts with metal nanoparticles, the positive core and negative electron cloud oscillate with some frequency resulting from the effect of the fluctuating nature of electric vector of the incident radiation and electrostatic coulombic attraction force to restore it. As the size of the nanoparticles reduces to the wavelength of light or smaller than that, the plasmon resonance become localized. If the incident radiation has the same frequency as the surface plasmon frequency of the nanoparticles, then there is resonance condition, and a maximum absorption of the corresponding light is observed, and this phenomenon is called surface plasmon resonance (SPR). Localized surface plasmon resonances (LSPRs) in metallic nanostructures significantly enhance light-matter interactions through subwavelength optical confinement, boosting the sensitivity of surface spectroscopies. While the dissipation of these surface plasmons limits field confinement, it also opens doors for photochemistry, photocatalysis, and photothermal heating.

S. Wu *et al.*^120^ examined the recent insights into the physics and dynamics of LSPR photothermalization, comparing steady-state behavior with ultrafast time-resolved studies to guide the optimization of plasmonic systems for applications under continuous, low-intensity illumination, like sunlight.

These luminescent materials also called photothermal agents are introduced in the body and they can circulate in the blood stream and accumulate or bind with the diseased area like a malignant tumor or cancer cells. These agents/nanoparticles not only help in the generation of heat but also in the mapping of the cancer cell. When an external light source, typically a near-infrared laser, irradiates the area, the photothermal agents absorb the light energy and efficiently convert it into heat. This localized heat increase rises the temperature of the targeted cells to a point where they are damaged or destroyed, while minimizing harm to the surrounding healthy tissue. PTT is particularly attractive due to its non-invasiveness, precision in targeting, and reduced side effects compared to traditional treatments like chemotherapy or radiotherapy. Various nanomaterials such as metal (Au, Ag, Pt, Cu, and Se) semiconducting (copper-based, zinc-based, and iron-based), carbon-based (carbon nanotubes and graphenes) and organic (organic dyes and semiconducting polymeric) are used as photothermal agents as per suitability and requirement.

PTT, sometimes also considered a form of localized hyperthermia, directly converts light energy into heat to ablate diseased cells. This therapy uses photothermal agents, often in the form of nanoparticles (*e.g.*, Au- and Ag-NPs), that efficiently absorb light, typically in the NIR spectrum, and then release this absorbed energy as heat. This localized heat elevation at the tumor site leads to the destruction of cancer cells. Unlike PDT, PTT does not require oxygen for its therapeutic effect and can often utilize longer wavelengths of light, which can penetrate deeper into tissues with less scattering and absorption by surrounding healthy tissues.

A promising cancer therapy involves the thermal ablation of diseased cells using individual or agglomerated nanoparticles as photothermal agents. This therapy functions by having the nanoparticles absorb photo-energy and convert it into heat, effectively destroying nearby biological media or cells. However, a crucial aspect of this method is the need to preserve surrounding healthy cells. Moreover, it was demonstrated that controlling the concentration of nanoparticle agglomerates can maintain the efficiency of the thermal agent while limiting the risk of damage to healthy surrounding tissues (T. Grosges *et al.*).^121^

Glutathione capped hollow Ag-NPs as a viable option due to utilizing non-toxic and inexpensive precursors was synthesized and synthesis parameters such as stabilizer type, reducing agent concentration, and reaction temperature was optimized by C. Bhavsar *et al.*^122^ The study further demonstrated the photothermally induced cytotoxicity of HAg-NPs in cancer cells using a 532 nm Nd:YAG laser, alongside their proven biocompatibility in both *in vivo* and *ex vivo* models, paving the way for future research into their *in vivo* antitumor activity and role in targeted drug delivery.

### 6.5 Bimodal or combined therapies

L. G. Arellano *et al.*^123^ introduced a novel hybrid nanosystem designed to simultaneously deliver both PDT and plasmonic PTT therapies to eliminate malignant cells. To achieve it, first Au nanorods were functionalized, layer-by-layer (LbL) assembly technique by introducing alternating layers of anionic poly (styrene sulfonate) (PSS) and cationic poly-l-lysine (PLL) as polyelectrolytes (PEs) and finally an outer layer of hyaluronic acid (HA) for colloidal stability and targeted delivery to CD44-overexpressing tumor cells. To enable PDT, NIR-sensitive photosensitizer indocyanine green was incorporated into the coating, resulting in PSS/PLL-ICG/HA-coated AuNRs. Under optimized NIR light excitation, these hybrid nanoparticles demonstrated significant ROS production (*ca.* 80% after 5 min) and exhibited substantial heating capabilities (9 to 22 °C temperature increments) due to ICG's light absorption contributing to the photothermal effect. *In vitro* studies at different power intensities and temperatures revealed that the combined PDT and PPTT effects resulted in up to 70% cell cytotoxicity, providing crucial insights into the optimal bimodal phototherapy conditions and the underlying cell death mechanisms.

Plasmonic photothermal therapy and drug delivery using resonant MNPs have emerged as promising selective cancer cell destruction techniques. A. H. Faid *et al.*^88^ utilized Au-NPs with high stability and a small size of 14 ± 4 nm and demonstrated significantly enhanced photothermal heating efficiency in MCF-7 breast cancer cells when combined with 532 nm laser irradiation. Furthermore, these Au-NPs proved effective as carriers for Dox, forming a DOX@AuNPs nanocomposite that showed increased cytotoxicity and a reduced IC50 against MCF-7 cells compared to free Dox.

Chemodynamic therapy (CDT) is a promising cancer treatment, with copper sulfide (CuS) nanostructures often used as Fenton-like reagents to generate toxic hydroxyl radicals from hydrogen peroxide (H_2_O_2_) (Z. Chen *et al.*).^124^ However, CDT's efficacy was limited by the tumor microenvironment (TME), including insufficient H_2_O_2_ and high glutathione levels. To overcome these limitations, researchers engineered a novel hollow CuS nanoplatform loaded with glucose oxidase (GOx) and catalase (CAT) and coated with macrophage membranes (M@GOx-CAT@CuS NPs). This biomimetic nanoplatform, guided by photoacoustic imaging, significantly enhanced ROS production and GOx catalytic activity *via* mild phototherapy, leading to a cascade of beneficial effects. In both *in vitro* and *in vivo* experiments, the M@GOx-CAT@CuS NPs demonstrated remarkable synergistic anticancer therapeutic effects, paving the way for more precise tumor diagnosis and treatment.

Bimetallic spherical nanocomposites ranging from 17.34 to 90.51 nm of nickel and zinc (Ni/Zn@orange/chitosan), utilizing an aqueous extract of *Citrus sinensis* and chitosan, were reported by J. Li *et al.*^125^ They demonstrated ability to remove Congo red and methyl orange dyes from aqueous solutions and efficiently uptake the drug mefenamic acid, and exhibited great photocatalytic activity in synthesizing benzimidazole. Moreover, they were proved to be potent electrochemical sensors for glucose and displayed acceptable anti-breast adenocarcinoma potentials against various malignant cell lines, highlighting their versatility as photocatalysts, dye removal agents, and promising new drugs for breast cancer treatment.

Quercetin was utilized by N. Karuvantevida *et al.*^126^ as an effective green reductant to synthesize size-controlled silver (QAg-NPs), gold (QAu-NPs), and bimetallic (QAgAu-NPs) nanoparticles. Notably, these flavonoid-functionalized noble metal nanoparticles exhibited significant bactericidal effects against both Gram-positive and Gram-negative pathogens and demonstrated promising cytotoxic potential against breast (MCF-7) and colorectal (HCT-116) carcinoma cell lines.

V. Garcés *et al.*^127^ developed magneto-optical hyperthermia agents as an innovative oral agent for hyperthermia cancer therapy by utilizing probiotic bacteria as a carriers for MNPs. Two distinct synthetic strategies were employed: one involved simultaneously loading *Lactobacillus fermentum* with magnetic nanoparticles (Mg-NPs) and Au nanoparticles, while the other, a novel approach, sequentially loaded AuNPs onto the bacteria followed by Mg-NPs-exopolysaccharides (EPS) to create a layered structure. These systems demonstrated the capacity to function either under alternating magnetic field or near-infrared laser light, which could serve for both kinds of cancer treatment such as magnetic hyperthermia and photothermal therapy. Given *Lactobacillus fermentum*'s proven ability as an oral drug carrier and its market presence as a gut microbiota supplement, these findings pave the way for developing new oral cancer treatments for gastric diseases using these advanced Au-NP/Mg-NP-bacterial systems.

Radiotherapy (RT) is a common and effective cancer treatment, and when combined with immune responses (radioimmunotherapy), it can partially inhibit distant and recurrent tumors. However, the effectiveness and safety of radioimmunotherapy are often limited by tumor radio-resistance and poor immunogenicity. To address these challenges, Y. P. Wang *et al.*^128^ introduced oxaliplatin(iv)-iron bimetallic nanoparticles (OXA/Fe NPs) as a cascade sensitizing amplifier for powerful, low-dose radioimmunotherapy. These OXA/Fe NPs accumulate specifically in tumors, where their released metallic components trigger cascade reactions that enhance RT by inducing DNA damage, boosting ROS and oxygen levels, and amplifying immunogenic cell death, ultimately leading to a robust immune response, effective inhibition of metastatic tumors, and long-term immunological memory.

## 7. MNPs in theranostics

Traditional cancer treatments like chemotherapy, radiotherapy, and surgery often come with significant drawbacks, including systemic toxicity from chemotherapeutic agents, damage to healthy tissues, and the risk of incomplete tumor removal or unnecessary tissue excision during surgery. The emerging field of nanotechnology offers a revolutionary approach to cancer management, particularly using MNPs that offer a new hope in diagnosis, with its ability to selectively target and control drug release, surpassing traditional treatments.

Theranostics, a combined approach of diagnostics and therapeutics, has become a crucial element in cancer treatment. Metal-based theranostic, emerging as a promising nanotechnology-based drug delivery system, offers numerous benefits in the treatment for deadly diseases like cancer, enabling early detection and precise medication monitoring, targeted drug delivery, and simultaneous diagnosis and treatment of early-stage cancer. The ability of theranostic nanoparticulate systems to aggregate at tumor sites facilitates noninvasive drug administration and provides valuable morphological and biochemical information about the tumor. T. G. Agnihotri *et al.*^129^ extensively explored recent advancements in metal-based theranostics, including MNPs, metal oxides, metal sulfides, and nanocomposites including challenges related to their clinical integration and toxicological aspects.

MNP-based improved treatment alternatives are emerging as a promising solution, offering unprecedented advancements in various biomedical applications, including cancer therapy, diagnosis, and imaging. The paper delves into the potential of nanoparticles made from metals, metal derivatives, hybrids, and alloys, discussing their diverse applications in cancer management, from delivering chemotherapeutics and vaccines to enabling ablative hyperthermia and theranostic platforms. D. N. Păduraru *et al.*^130^ discussed metal-based nanoparticles that are most frequently utilized in cancer applications, highlighting the advantageous properties of various nanoscale metals.

Moreover, the plant-based metal nanoparticles are believed to be potential antioxidants and cancer-fighting agents, making their biosynthesis and applications in cancer theranostic a promising area for future research (K. Gulia *et al.*).^131^Among all MNPs, Au-NPs stand out due to their high biocompatibility, non-toxicity, ease of synthesis, and effective functionality. This innovative technology of theranostics provides a dual advantage: enabling precise fluorescent bioimaging and facilitating targeted drug delivery to specific tissue sites, thereby minimizing negative impacts on surrounding healthy areas and promising to elevate current diagnostic and treatment practices while reducing patient stress (S. Kulkarni *et al.*, 2022).^132^

A highly efficient gold nanostar@polyaniline (AuNS@PANI) core–shell nanocomposite designed for photoacoustic imaging (PAI)-guided photothermal therapy (PTT) is reported by Y. Wang *et al.*^133^ By optimizing the composition, this nanocomposite achieves an impressive photothermal conversion efficiency (PCE) of up to 78.6%, significantly outperforming individual components and most existing agents. Modified with hyaluronic acid (HA) for tumor targeting, these nanoprobes (AuNSPHs) demonstrate promising biocompatibility and effectively suppress triple-negative breast cancer growth in both *in vitro* and *in vivo* studies with a low gold dosage. This novel theranostic agent leverages its superior photothermal conversion ability to enhance the antitumor efficacy, showing great potential for future medical applications.

## 8. Antimicrobial/antiviral/antibacterial and antifungal applications of MNPs

MNPs are gaining significant attention in the fight against microbial infections, particularly due to the growing problem of antibiotic resistance. Their unique properties at the nanoscale allow them to interact with microbes in ways that conventional antibiotics often cannot, making it more difficult for bacteria to develop resistance. MNPs exhibit multiple, simultaneous mechanisms of action against microbes, which is a major advantage over traditional antibiotics that often target a single pathway. These mechanisms include:

(a) Membrane disruption: most of the MNPs due to the positive surface charge in contrast to the bacterial cell walls, which are typically negatively charged, can disrupt the membrane *via* strong electrostatic interaction, forming pores or holes in the membrane, which leads to increased permeability, and hence, leakage of essential intracellular components such as ions, proteins, and genetic materials, ultimately causing cell death.

(b) ROS generation: MNPs can induce oxidative stress within microbial cells by generating ROS (*e.g.*, superoxide ions, hydrogen peroxide, and hydroxyl radicals), which can cause severe damage to vital cellular components such as DNA, proteins, lipids, and enzymes, impairing metabolic pathways and leading to cell death.

(c) Metal ion release: MNPs in the vicinity of the membrane release metal ions which are highly reactive and can interfere with various cellular processes, including enzyme function, protein synthesis, DNA replication, and respiratory chain activity, and inhibit cell wall synthesis.

(d) Damage to biomolecules: beyond ROS generation and ion release, nanoparticles can directly interact with and damage essential biomolecules like DNA and proteins within the microbial cell, leading to impaired replication, transcription, and overall metabolic activity besides the disruption of ATP production, which is critical for bacterial survival.

**Fig. 6:**
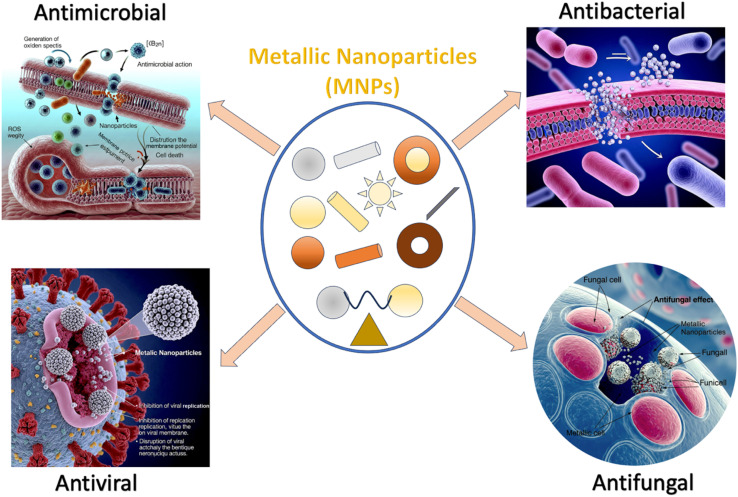
Antimicrobial/antiviral/antibacterial/antifungal applications of different types of MNPs. Figure partially created using Google Gemini.

The processes of antimicrobial/antiviral/antibacterial/antifungal applications of different types of MNPs are shown and described in Fig. 6.

### 8.1 Antimicrobial

Commonly used MNPs for antimicrobial applications are Au, Ag, Cu/CuO, Zn/ZnO, Fe/iron oxide nanoparticles, *etc.* The multifaceted antimicrobial activity of MNPs makes them promising not only for antimicrobial coatings of medical devices (implants and catheters) and wound dressings but also for efficient drug delivery systems, which can carry antibiotics or other antimicrobial agents by enhancing their solubility, stability, and targeted delivery action.

Nanomaterials can generate ROS due to defect-induced electronic interactions with oxygen and water, leading to significant pro-oxidant activity influenced by environmental and structural factors (S. Podder *et al.*).^134^ This pro-oxidant activity is evident in their antibacterial effects under various conditions, such as different light levels. Conversely, some nanomaterials also exhibit a scavenging phenomenon, demonstrating antioxidant activity by inhibiting ROS, which is also linked to their electronic properties. The ability of these nanomaterials to switch between pro-oxidant and antioxidant activities, depending on factors like concentration and morphology, suggests their promising potential for applications in cancer therapy and inflammatory disease treatment also.

Ag-NPs derived from the *Neurada procumbens* plant exhibited significantly (*p* ≤ 0.05) stronger antibacterial activity against Gram-positive strains (*S. aureus* and *B. cereus*) compared to *E. coli*. Overall, the biosynthesis of Ag-NPs using *Neurada procumbens* extract presented an ecofriendly method for creating nanostructures with promising antimicrobial and anticancer properties.

Ag-NPs synthesized by utilizing *Alhagi persarum* flower (APF) extracts as both the reducing and capping agents revealed that 18 bioactive compounds, including flavonoids and phenolic acids, from the APF extract were responsible for the reduction and stabilization of the Ag-NPs. These biosynthesized Ag-NPs exhibited significant antimicrobial activity against multidrug-resistant bacterial and fungal pathogens. Moreover, the Ag-NPs demonstrated promising antioxidant activity and cytotoxicity against human breast adenosarcoma cells (MCF-7), suggesting their potential as a therapeutic formulation for bacterial infections and breast cancer (S. Korpayev *et al.*).^135^

The eco-friendly synthesis of MNPs using biological materials represents an encouraging and innovative approach in nanotechnology, favorable for its high efficiency and purity. C. Keskin *et al.*^136^ synthesized spherical and an average size of 27.12 nm Ag-NPs in a rapid and simple manner using an aqueous extract from green *Diospyros kaki* L. (DK) leaves, which exhibited strong antibacterial activity against both Gram-positive and Gram-negative human and foodborne pathogens, along with good antifungal activity. Furthermore, it demonstrated dose-dependent cytotoxic effects on glioblastoma, human colorectal adenocarcinoma, and human ovarian sarcoma cancer cell lines, notably inhibiting CaCo-2 cell viability by up to 59.49% at 50 µg mL^−1^, highlighting their potential as effective antibacterial and prospective anticancer agents.

The rise of multidrug-resistant pathogens and the slow progress in developing new anticancer and antimicrobial drugs are major global health concerns, intensified by the limitations of current treatments. Ag- and Au-NPs were synthesized using the highly productive fungus *Trichoderma saturnisporum*, and Ag-NPs were particularly effective against pathogenic bacteria, including MRSA, while Au-NPs showed stronger antibiofilm activities. Both types of NPs demonstrated antioxidant properties and promising cytotoxicity against MCF-7 cancer cells, alongside low toxicity to normal Vero cells.^137^

Small size (around 19.40 nm), spherical and polydispersed Ag-NPs were fabricated by using the potent *Amycolatopsis* sp. strain MN235945, which exhibited significantly increased antimicrobial activity and effectively inhibited cervical adenocarcinoma HeLa cells (IC50: 81.12 µg ml^−1^) and human breast cancer MCF-7 cells (IC50: 235.45 µg ml^−1^).

*Thuja occidentalis* recognized for its traditional use in treating skin disorders was also explored by R. Yu *et al.*^138^ for the synthesis of stabilized Ag-NPs (ranging from 40 to 80 nm in size) and their potential in nanoparticle-based wound healing. These Ag-NPs demonstrated cytotoxic effects on MCF-7 cancer cells while exhibiting a low toxicity profile with an LD_50_ greater than 3000 mg kg^−1^. Importantly, the Ag-NPs significantly promoted wound healing in patients who underwent anorectal surgery, leading to complete lesion closure by the 17th day and suggesting their promise as a safe, efficient, non-toxic, and eco-friendly wound dressing material for chronic wounds.

*Berberis vulgaris* extract was utilized for the synthesis of Ag-NPs with optimal production achieved by controlling various reaction parameters by Z. Hashemi *et al.*^139^ These synthesized NPs were of oval and spherical shape with an average crystalline size of 45–60 nm and demonstrated exceptional photocatalytic efficiency by degrading 97.82% of methyl orange under sunlight in a short timeframe. Furthermore, they exhibited potent antibacterial activities against seven clinically isolated multidrug-resistant bacteria and significant cytotoxic effects on human gastric cancer (AGS) and breast cancer (MCF-7) cell lines, highlighting their promising potential for both industrial wastewater treatment and diverse biomedical applications.

Q. Maqbool *et al.*^140^ utilized *Olea europaea* leaves for Cu-NPs, and their efficacy was evaluated in treating ovalbumin-induced allergic conjunctivitis likely stemming from their anti-inflammatory properties. Impressively, treatment with various doses of the Cu-NPs significantly reduced clinical scores of allergic conjunctivitis and decreased ovalbumin-specific IgG and IgE, as well as total serum IgE levels.

Se-NPs being particularly notable for their biocompatibility, stability, and low toxicity were synthesized using an aqueous extract from the discarded colored outer skin of *Pistacia vera* L. (Siirt pistachio), a waste product rich in bioactive compounds (M. F. Baran *et al.*).^141^ Comprehensive characterization confirmed the formation of highly distributed, spherical, and monodisperse Se-NPs with an average size below 10 nm and revealed that functional groups from the plant extract were crucial for their capping and bio reduction. Significantly, these biogenic Pv-Se-NPs exhibited antimicrobial activity comparable to standard antibiotics and demonstrated potent dose- and time-dependent cytotoxic effects against cancer cell lines. These findings highlight the considerable potential of Pv-SeNPs for developing antimicrobial and/or anticancer medications across pharmaceutical, cosmetic, and food industries. The trigonal and semi-spherical Se-NPs with a metallic core and capped with a bioactive compound were synthesized using a *Crocus caspius* aqueous extract by S. R. Alizadeh *et al.*^142^ These biosynthesized Se-NPs exhibited considerable antioxidant, antibacterial, and antifungal activities, alongside a strong growth inhibitory effect on MCF-7 and AGS cancer cells, and demonstrated antileishmanial activity against promastigotes.

### 8.2 Antiviral applications

Viruses are one of the main agents of various diseases. MNPs are being utilized as antiviral agents; for instance, intracellular Ag-NPs synthesized *via Aspergillus ochraceus* have shown significant antiviral potential.^143^ The feasibility of the mycosynthesis of Ag-NPs and their antiviral activity differs with the production system used, which has been studied by NPs.^144^ The authors explained the interactions of Ag-NPs with human parainfluenza virus type 3 and herpes simplex virus types 1 and 2, which are dependent on the size of Ag-NPs. In this research, the diminution of viral infectivity has recognized the potential of Ag-NPs to obstruct the interaction between the virus and the cell, which might be dependent on the zeta potential and size of the Ag-NPs (Galdiero *et al.*).^144^

Ag-NPs can act as potent antiviral agents against arenaviruses by inhibiting the replication of the Tacaribe virus, a type of arenavirus, even though they initially enhanced the virus's entry into host cells. The inhibitory action targets the early stages of viral replication, as their effectiveness was highest when applied before or immediately after infection. Moreover, this inhibitory effect led to a significant decrease in the virus's ability to produce new RNA and release of new viral particles (J. L. Speshock *et al.*).^145^

Ag-NPs are reported by H. H. Lara *et al.*^146^ to exhibit antiviral properties against various HIV-1 strains at concentrations that are not harmful to cells. These nanoparticles act as virucidal agents by binding to the gp120 protein on the virus, which prevents it from attaching to host cells. The effectivity of the nanoparticles was obtained by blocking the viral entry and inhibiting the later stages of the viral life cycle.

V. J. Rogers *et al.*^147^ reported Ag-NPs to inhibit monkeypox virus (MPV) infection *in vitro* without causing harm to host cells. Nanoparticles around 10 nm and silver nitrate were effective at reducing the number of viral plaques in a dose-dependent manner.

Despite the proven efficacy against HIV and HBV, AgNPs with a diameter 10 nm demonstrated anti-H1N1 influenza A virus activity. Electron microscopy and flow cytometry showed that the nanoparticles can reduce the apoptosis (cell death) caused by the virus in infected cells.^148^

### 8.3 Antibacterial applications

In recent years, the intersection of cancer treatment and bacterial infection has gained significant interest in antibacterial nanosystems capable of loading and delivering anticancer agents. In the pursuit of next-generation nanomedicine, theranostic nanotherapeutic strategies are gaining prominence; unlike current agents, these strategies can eradicate drug-resistant strains and cancerous cells while also preventing their formation.

When bacteria are exposed to Ag-NPs, cytotoxicity occurs. The silver ions and silver NPs interact with the plasma membrane, which results in the irritation of cellular respiration, cell permeability, and prevention of DNA replication by denaturing the ribosomes or by binding the DNA (T. C. Dakal *et al.*).^149^ In addition, the mechanism of Au-NP-induced antibacterial potential against *E. coli* is studied and explained by Cui *et al.* The authors stated that gold NPs distorted the membrane potential and inhibited the activity of ATPase, resulting in a significant decrease in cellular ATP (Cui *et al.*, 2012).^150^ The exposure to Au-NPs showed inhibition of rRNA binding with ribosomes. The antimicrobial potential of bacteria-mediated synthesized Ag-NPs and Au-NPs has also been studied.^151,152^ In a different study, *Streptomyces hygroscopicus*-mediated biologically synthesized NPs were used as antimicrobial agents *versus Enterococcus faecalis*, *Bacillus subtilis*, *Staphylococcus epidermidis*, *E. coli*, *S. aureus*, and *Salmonella typhimurium*. Recently, *Saraca asoca* plant leaf extract was used for the synthesis of Ag-NPs, and the antibacterial potential of synthesized NPs was investigated against *Salmonella typhi Staphylococcus aureus*, and *Streptococcus pyogenes*.

Ag-NPs were synthesized by P. Kaur *et al.*^153^ utilizing *Laurus nobilis* leaf extract, which possessed a face-centered cubic crystal structure with a mean particle size of 13 ± 4 nm. Moreover, it exhibited significant *in vitro* antibacterial activity against *Staphylococcus aureus* and *Escherichia coli* and demonstrated high cytotoxic activity against human liver cancer cells with an IC_50_ value of 8.86 ± 0.74 µL mL^−1^.

Ginger-based Ag-NPs were synthesized by S. Mehrotra *et al.*^154^ and utilized as an effective and promising strategy for cancer treatment and the prevention of bacterial infections. Leveraging ginger's well-known medicinal properties and phytochemicals like gingerols and shogaols, Ag-NPs were biosynthesized from an ethanolic ginger rhizome extract, yielding spherical nanoparticles in the 80–100 nm range, which demonstrated antioxidant and antibacterial activities against several Gram-positive and Gram-negative bacterial species. It also exhibited promising cytotoxicity against Vero cell lines, indicating the potential for these non-toxic and eco-friendly nanomaterials in developing broad-spectrum antimicrobial and anticancer medications.

J. Xu *et al.*^155^ demonstrated the green synthesis of non-toxic, stable, spherical shaped and small-sized (ranging from 3.91 to 27.07 nm) Ag-NPs using *Solanum tuberosum* peel extract, which significantly inhibited the growth of several bacterial strains and *Candida albicans*. Furthermore, *in vitro* analysis revealed that these Ag-NPs exhibited dose-dependent cytotoxicity against human glioblastoma, colorectal adenocarcinoma, and ovarian cancer cell lines, indicating their considerable potential as both antibacterial and anticancer agents for biomedical and industrial uses.

While Ag-NPs are widely recognized for their broad-spectrum antibacterial activity, their inert surfaces typically hinder direct drug loading. W. Ha *et al.*^156^ studied the biocompatible tannic acid-modified TAAg-NPs, synthesized using tannic acid as a green reducing and stabilizing agent, which demonstrate low cytotoxicity to normal cells and potent antibacterial effects against *E. coli* and *Staphylococcus aureus*. Crucially, these TAAg-NPs effectively load and deliver the anticancer drug epirubicin hydrochloride (EPI) *via* strong electrostatic interactions, exhibiting pH- and glutathione-sensitive drug release. The resulting TA-AgNP/EPI nanodrug maintains equivalent cytotoxicity to free EPI against Hep G2 cells and, more importantly, effectively mitigates the negative influence of bacteria on EPI's cytotoxicity, showing promise for both tumor suppression and anti-infection in animal models.

*Polianthes tuberosa* flower extract was utilized to synthesize PTAg-NPs, which exhibited dose-dependent bactericidal activity against *E. coli* and *S. aureus*, and demonstrated significant dose-dependent toxicity against the A431 skin cancer cell line with an IC50 of 54.56 µg mL^−1^ (Alghuthaymi *et al.*, 2023)^157^. Furthermore, the PTAg-NPs induced DNA damage, increased ROS, and triggered apoptosis in melanoma cells, suggesting their potential as a targeted therapy for skin cancers without harming normal tissues.

MNPs, particularly Ag-NPs, have shown significant promise in biomedical applications, and their inclusion within polymeric matrices like dendrimers through chemical functionalization is proven by their high effectiveness. M. K. Rai *et al.*^158^ highlighted the growing trend of developing Ag-dendrimer nanocomposites, capitalizing on silver's established antibacterial activity and dendrimers' unique properties, for diverse bactericidal and biomedical applications in the pharmaceutical sector.

The rise of multidrug-resistant (MDR) bacteria poses a significant challenge to medicine, increasing patient mortality, morbidity, and the cost of treatment. In response, there is an urgent need to develop new antimicrobial agents that can combat these resistant microbes. AgNPs have emerged as a promising powerful “nanoweapon” for both treating and preventing infections caused by drug-resistant bacteria (X. Fu *et al.*).^159^

### 8.4 Antifungal applications

Recently, studies have been performed on biologically synthesized MNPs for their antifungal properties. *Alternaria alternata*-mediated extracellularly synthesized biological Ag-NPs showed increased antifungal activity of fluconazole against *Fusarium semitectum*, *Phoma glomerata*, *Candida albicans*, *Trichoderma* sp., and *Phoma herbarum* (M. Gajbhiye *et al.*, 2009).^97^ The synthesized Ag-NPs extracted from *Ocimum vasilicum* leaves were applied as antibacterial as well as antifungal agents, and it has been shown that they have higher antifungal activity against *Aspergillus flavus*, *Aspergillus niger*, *Aspergillus terreus*, and *Aspergillus fumigatus* (Elumalai D. *et al.*, 2019).^99^

M. C. Mkouboi *et al.*^160^ investigated sixteen different silver- and zinc-based nanoparticles using *Cephalaria tchihatchewii via* phytobiological methods, resulting in nanoparticles ranging from 50 to 297 nm in diameter. The cytotoxic activities of these nanoparticles were evaluated against the human lung (A549), breast (MDA-MB-231), and prostate (PC3) cancer cell lines, as well as healthy lung (CCD-34Lu) cells. Notably, silver nitrate and loganin-Ag-NPs showed significant inhibition against PC3 cells, with IC50 values of 2.39 ± 0.16 and 7.84 ± 1.21, respectively, outperforming doxorubicin (12.99 ± 1.57 µg mL^−1^). These findings suggest that *Cephalaria tchihatchewii* is a promising candidate for various biopharmaceutical applications, particularly anticancer therapies.

The fabrication of MNPs through green synthesis has significantly influenced global research toward eco-friendly practices, marking a “green revolution” in science and technology. S. E. Arland *et al.*^161^ investigated the synthesis of Ag-NPs with irregular morphologies and a polycrystalline nature *via* both chemical co-precipitation and using *Cassia fistula* L. flower extract. The green-synthesized Ag-NPs demonstrated superior antioxidative (84.48%) and anti-inflammatory activities (IC50 of 30.19 µg mL^−1^) compared to the chemically synthesized Ag-NPs. Furthermore, the green-synthesized Ag-NPs showed more pronounced dose-dependent cytotoxicity against HEP3B cancer cells *in vitro*.

## 9. Biomedical imaging

**Fig. 7:**
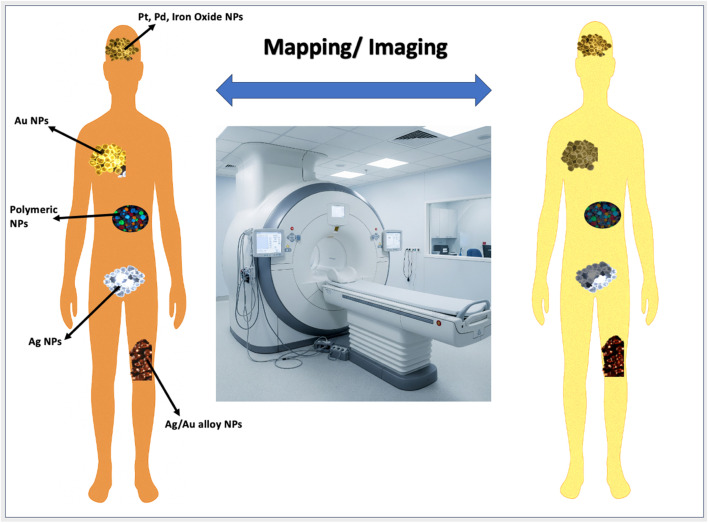
Representative image of the different types of luminescent MNPs and their applications in mapping/imaging of various cancers. The applications of these nanoparticles are not limited to the specific cancers shown in the image; rather, they extend to a diverse range of other cancer types. Figure partially created using Google Gemini.

MNPs have been appealing for imaging and spectroscopic analysis of biological samples because of their unique optical properties caused by localized plasmon resonance (M. C. Mkouboi *et al.*).^160^ A representative image of the different types of luminescent MNPs and their specific applications in the mapping/imaging of various cancers are shown in Fig. 7. However, their applications are not limited to the specific cancers mentioned in the image; rather, they are more diverse. For example, gold (Au) and silver (Ag) nanoparticles are widely utilized for the detection and treatment of various cancer types. Au-NPs are broadly used as optical contrast agents for bioimaging since they possess several advantages such as strong light scattering properties at visible wavelengths without photobleaching, high chemical stability, and low toxicity for use in biological samples. Current research has revealed high-spatiotemporal resolution optical imaging of Au-NP-labeled biomolecules and intracellular organelles.^162,163^ In addition to their use as contrast agents, MNPs also act as probes for surface-enhanced Raman scattering (SERS) spectroscopy to investigate biological molecules and cells (J. Kneipp *et al.*, 2007).^164^ Information such as high sensitivity and high spatial confinement of the molecular species near the surface of the metal can be revealed by SERS spectroscopy. This allows the analysis of biological functions and phenomena, such as organelle transportation (C. A. R. Auchinvole *et al.*),^165^ drug uptake (Y. Furuya *et al.*),^166^ and cell division (J. Conde *et al.*).^167^ When labeled with Au-NPs, the movements of biomolecules and intracellular organelles can be explored by tracking the bright spot of the Au-NP scattering image. The localization precision of the optical image is inversely proportional to the square root of the photon number.^166^ Au-NPs provide a high scattering signal without photobleaching, allowing for nanometer-scale localization even at microsecond time resolutions. In addition, Au-NPs enable the evaluation of the fast dynamics of biomolecules such as lipids and proteins. Additional enhancements in localization precision are important to comprehend the working mechanism of such tiny and complex biological molecules in detail. Recent research described the development of an annular illumination total internal reflection dark-field microscope to illuminate gold NPs at a high laser intensity (Conde, Rosa, *et al.*, 2012).^167^ The application of small Au-NPs as optical probes is essential to reduce possible steric hindrances on the target biomolecules. Recently, the development of extremely sensitive interferometric scattering microscopy achieved localized precision at a few nanometers with 20 nm gold NPs at microsecond time resolution (P. V. Baptista *et al.*).^168^ Detection using Au-NPs with smaller diameters, such as 10 nm (H. M. E. Azzazy)^169^ or even label-free direct detection of proteins (R. A. Sperling *et al.* and P. V. Baptista *et al.*),^168,170^ has also been performed, enlarging the range of measurable biomolecule types and phenomena. However, emerging methods now enable the simultaneous analysis of multiple biomolecules at high spatiotemporal resolution by utilizing additional color channels. In other words, multicolor imaging of biomolecules with silver, silver/gold alloy, and gold NPs is not possible (E. Boisselier *et al.*).^171^ A multicolor high-speed single-particle tracking system has been recently developed using silver and silver–gold alloy nanoparticles together with Au-NPs (R. A. Sperling *et al.*, 2008).^170^ Another similar application of MNPs is their wide-ranging usage as SERS probes to amplify the Raman scattering signal from molecules near the metal surface, enabling highly sensitive Raman measurements of biomolecules such as DNA, RNA, amino acids, proteins, and lipids.^172,173^ The introduction of MNPs into cells allows them to work as SERS probes that report the molecular and chemical environment in cells near the metal surface (T. K. Sau *et al.*).^174^ One of the important applications of SERS is the detection of extrinsic molecules such as drugs introduced into cells. SERS detection of anti-tumor and anti-leukemia drugs in cells has revealed much about drug distribution and metabolism in cells (C. J. Murphy *et al.*, 2005)^175^ (A. R. Kherlopian *et al.*).^176^ Another application of SERS with metallic NPs is a sensing method for intracellular environments such as pH and redox potential. For this reason, the nanoparticle surface is functionalized with molecules that show a structural change depending on the surrounding environment. Specifically, SERS-based pH sensing of cells has been widely examined,^177–179^ as the pH value is related to various biological processes such as proliferation, apoptosis, and ion concentration.^180,181^

F. Mansoor *et al.* (2023) introduced a novel approach for synthesizing highly advanced cubic Ag nanomaterials, which serve as sacrificial templates for creating Au nanocubes.^182^ The Ag nanocubes were initially formed using sodium thiosulphate and KCl, with the study evaluating the impact of various potassium halides on their seed-mediated growth to ensure precise morphological control. Leveraging their dual metallic properties, Ag@4MBA@AuNCs nanostructures were synthesized**,** incorporating 4-mercaptobenzoic acid (4MBA) as a Raman reporter molecule. This engineered nanostructure demonstrated an extraordinary 1010-fold enhancement in surface-enhanced Raman scattering (SERS) signals, enabling highly sensitive imaging capable of detecting even three breast cancer cells (MCF-7 cells) *in vitro*, while also facilitating the catalytic reduction of toxic nitrophenol.

Oral cancers, a type of head and neck squamous cell carcinoma, present significant therapeutic challenges, making effective and non-invasive treatments like radiotherapy crucial. S. Ahmad *et al.*^183^ focused on synthesizing one-pot PEGylated Au-NPs and Ag-NPs to assess their toxicity and radio sensitization effects in the oral cancer KB cell line. Both types of nanoparticles exhibited a near-spherical morphology and a hydrodynamic diameter of approximately 50 ± 5 nm, with Au PEG NPs demonstrating superior CT contrast enhancement compared to clinically used agents and Ag PEG NPs. Notably, Au PEG NPs showed a higher radio sensitization effect than Ag PEG NPs in the KB cell line, suggesting their strong potential as both an enhanced radiosensitizer and a CT imaging agent for oral cancers.

### 9.1 MRI and computed tomography (CT)

Biological imaging is a rapidly developing field in fundamental biology and medical science as well (Z. Hashim *et al.*, 2014).^177^ The usage of nanoparticles as imaging probes has several benefits over conventional imaging agents. One of these advantages is loadability, where the concentration of the imaging agent can be monitored within each nanoparticle during the synthesis process. The tunability of the surface of nanoparticles that can potentially broaden the circulation time of the agent in the blood or target a specific location within the body is another advantage. Ultimately, nanoparticles can behave as multifunctional MI agents, since they have two or more properties that can be used simultaneously in multiple imaging techniques, and especially in MRI (C. Corot *et al.*).^178^ Metallic NPs are also used as tracers or contrast agents for a variety of medical imaging methods, such as fluorescence imaging, photoacoustic, and MRI. Iron oxide nanoparticles have been FDA-approved for MRI since the mid-1990s (D. P. Cormode *et al.*).^179^ Generally, nanoparticles carry a high payload of contrast-generating materials compared with small molecules, which gain more interest in imaging domains (Medintz *et al.*, 2005).^184^ In addition, NPs such as iron oxides or quantum dots yield contrast that cannot be produced by small molecules [(Corot *et al.*, 2006a), (Cormode *et al.*, 2009), (Moghimi SM *et al.*, 2001), (Medintz *et al.*, 2005)].^178–180,184^ The properties of the coating material of nanoparticles allow them to have long blood circulation half-lives (W. J. Mulder *et al.*).^185^ In addition, numerous properties/components can be included in nanoparticles with relative ease (D. Kim *et al.*, 2007).^186^ Nanoparticles in biomedical settings are now being applied to the development of contrast agents for computed tomography (S. Mukundan *et al.*, 2006).^187^ CT contrast agents that flow for a long time (D. R. Arifin *et al.*, 2011)^188^ can be used in applications where repeated injection of iodine contrast agents is needed, such as for stent placement. Nanoparticles can be used to allow cell tracking *in vivo* (Cormode *et al.*, 2010).^189^ Targeted nanoparticle CT contrast agents can identify the expression of proteins or cell types in tissues, for example, detecting the macrophage content of atherosclerotic plaque (Schlomka *et al.*, 2008).^190^ Nanoparticles can also be used with promising CT techniques, such as spectral CT, which can specifically detect exogenous contrast agents (M. M. van Schooneveld *et al.*, 2010).^191^ Moreover, nanoparticles can be multifunctional and so can provide contrast for multiple imaging modalities, for example, MRI, CT, and fluorescence (G. von Maltzahn *et al.*, 2009),^192^ or provide therapeutic effects as well as CT contrast (F. Stacul *et al.*, 2011).^193^ Nanoparticles may be compatible with patients for whom conventional iodinated contrast media are contraindicated due to renal deficiency or allergic responses (Rabin *et al.*, 2006).^194^ Core-based NPs such as metals, metal alloys, or metal salts are the main NPs that are studied for CT [(Q. Cai *et al.*, 2007, and Mukundan *et al.*, 2006)].^187,195^ In short, enhanced blood pool agents have been developed, new elements have been introduced (*e.g.* tantalum, gold, and bismuth), targeted and cell tracking agents have been reported and specific detection with spectral CT is now possible. Several advanced blood pool agents, such as long-circulating iodine-loaded liposomes and small, renally excretable gold or tantalum oxide nanoparticles are nearing clinical trials [(J. F. Hainfeld *et al.*, 2006), and (P. J. Bonitatibus *et al.*, 2012)].^196,197^

Besides drug delivery, MNPs are utilized in cancer detection tools like magnetic resonance imaging (MRI) and as colloidal mediators for magnetic hyperthermia. Their advantages including biodegradability, biocompatibility, high drug payload, and low toxicity make MNPs a promising avenue for improving skin cancer treatment, especially when combined with controlled-release systems stimulated by various external factors.

Recently, X-ray computed tomography (XCT) and X-ray fluorescence (XRF), which are complementary, non-invasive, nanometer-resolution, imaging techniques, are being utilized to study cellular structures and map multiple chemical elements and compositions to understand drug delivery and cellular uptakes by cancer cells.^198,199^ Metallic Ru and rhodium (Rh) nanoparticles are mostly used as contrast agents to utilize as probes for bioimaging with these techniques. Nevertheless, Ru and Rh nanoparticle size ranges of 1–3 nm and 6–9 nm, respectively, exhibited minimal toxicity against macrophages and ovarian cancer cells.^200^ Yuyang Li, *et al.*^201^ have reported the synthesis characterizations and use of various potent nanoparticles as contrast agents for XRF and XCT techniques to utilize for various bioimaging modalities. These advanced techniques, which offer significant advantages over conventional methods, are set to become a milestone in the future of diagnosis, mapping, and treatment for cancer and other diseases.

## 10. Biosensing

Recently, nanomaterials have been extensively exploited in the area of biosensing, particularly MNPs, due to their special characteristics. The unique properties of noble metal NPs allowed the development of new biosensing platforms with enhanced capabilities in the specific detection of bio-analytes (W. J. Mulder *et al.*, 2007).^202^Table 2 illustrates the various techniques used for biosensing applications of metal nanoparticles (Malekzad *et al.*, 2017).^203^ The simplicity of functionalization *via* simple chemistry and high surface-to-volume ratios among other unique physiochemical properties aligned with other optical properties of noble metal NPs have triggered the development of a plethora of biosensing platforms.^204^ Such unique physiochemical properties of noble metal NPs have led to the development of a wide range of biosensors such as (1) nanoprobes for *in vivo* sensing/imaging, cell tracking and monitoring disease pathogenesis or therapy monitoring and (2) nano-biosensors that are used for point-of-care disease diagnosis in addition to other nanotechnology-based tools that are beneficial for scientific research on basic biology [(D. Kim *et al.*, 2007), (Mukundan *et al.*, 2006), (Arifin *et al.*, 2011), (Schlomka *et al.*, 2008)].^186–188,190^ Noble metal NPs are the most conventional nanotechnology-based approaches for developing biosensors. This is due to their physiochemical malleability, high surface areas, and simplicity.^191^ Chemical methods such as thermal decomposition, co-precipitation, hydrolysis, photochemical reduction and chemical reduction, among other techniques, are used to synthesize noble metal NPs. In addition, physical methods include grinding, laser ablation, and vapor deposition. The goal is to obtain NPs with a good level of homogeneity and provide fine control over surface properties, sizes, and shapes, to better take advantage of their unique physicochemical properties for biosensing (von Maltzahn *et al.*, 2009).^192^ Due to their unique optical properties and ease of derivatization with different biomarkers in aqueous solutions, Au-NPs are the nanoparticles widely used in biosensing applications [(Stacul *et al.*, 2011), (Rabin *et al.*, 2006), (Chou *et al.*, 2010a)].^193,194,204^ The synthesis of GQDS/Ag NP hybrid structure and their use as a colorimetric glucose detector based on the color change process of the hybrid are also reported by S. Chen *et al.*, 2014.^205^

**Table 2:** Biosensor types, relevant transduction pathway, common techniques employed for each transduction pathway, and prevalent bio-recognizing agents for each type^186–188,190–192,196,197,202–205^

Biosensor according to transducer type	Type of change detected	Biological recognizer element	Common techniques
Calorimetric biosensors	Temperature changes	Enzymes	Thermistors
Optical (opto-electric) biosensors	Fluorescence/absorbance and other optical properties	Microbial cells	Colorimetric
		Nucleic acids	Fiber optics
		Antibody	SPR
		Aptamer	Light addressable potentiometric
		Enzyme	
Piezoelectric	Change in resonant frequency of crystals due to change in mass	Antibody	Surface acoustic wave
		Nucleic acid (DNA, RNA)	Surface transverse wave
			Crystal resonance frequency
Electrochemical	Redox reaction/electrical conductivity changes due to a change in ion concentration	Microbial cells	Conductometric
		Aptamer	Potentiometric
		Antibody	Amperometric
		DNA	
		Enzyme	

## 11. Limitations of MNPs within biomedical applications and clinical trials

Although numbers of MNPs have been identified and being utilized for biomedical applications but still further studies on their optimization of exposure time, potential biological toxicity, long term health risk are in incubation and required to be investigated and evaluated thoroughly before clinical trial (N. Santos *et al.*, 2025).^206^ The toxicological effect depends on the type, shape, size, surface functionalization, and exposure methods, which can cause not only inflammation, oxidative stress, genotoxicity, and cytotoxicity but also their distribution and accumulation in different parts of the body such as liver, spleen, and brain and can increase their toxicity and decrease biocompatibility, finally resulting in severe negative health impacts.

Among the extensively utilized MNPs, the toxicological effects of Ag, Au, iron oxide, ZnO and TiO_2_ NPs and their clinical trial overviews are well addressed by R. Krishna *et al.*^207^ Regarding the cytotoxicological assessment of Ag-NPs, some of the *in vivo* and *in vitro* studies revealed that they can easily accumulate in various tissues such as ilium, liver and kidneys with persistent accumulation in the brain and testis, causing ROS generation, oxidative stress, DNA damage and genomic toxicity.^208,209^

Although Au-NPs exhibit cytotoxic effects similar to Ag-NPs at higher concentrations, these effects can be mitigated through suitable functionalization. Such modifications significantly reduce toxicity and enhance overall biocompatibility.^210,211^ Lower doses of Au-NPs (<400 µg Au per kg) showed no untoward effects; however, higher amounts exhibited toxicity, depending on the route of administration and particle size. In addition, biocompatibility and toxicity depend on the choices of the cell and target tissues. For example, AuNPs showed dose-dependent sensitivity of spermatozoa in contrast to no noticeable effects on embryos after exposure.^212^ The safe and effective clinical integration of copper-based nanoparticles requires overcoming hurdles in biocompatibility and controlled release—areas thoroughly assessed in the work of M. J. Woźniak-Budych *et al.*^213^ As an alternative to MNPs, the bi metallic nanoparticles which show improved antimicrobial activity and reduced toxicity are also evaluated from the point of view of toxicity. The cytotoxicity of AuPt bimetallic nanoparticles was assessed by D. M. D. Formaggio *et al.* through *in vitro* assays on the HS68 human fibroblast cell line and *in vivo* embryonic toxicity tests in zebrafish (*Danio rerio*).^214^ The adverse effect of the MNPs on the central nervous system, which is one of the most delicate parts of the human body, is evaluated by K. Sawicki *et al.*^215^ by the modes of exposure to understand the mechanism of neurotoxicity.

A variety of advanced strategies such as surface modification by utilizing distinct methods of functionalization, suitable nanoparticle targeting approaches and morphology modifications, are required to reduce toxicological risks and for preclinical and clinical translations (García-Torra *et al.*, 2021).^216^

## 12. Conclusions and future aspects

### 12.1 Conclusions

Nanotechnology is an emerging field of biomedicine. MNPs have a variety of biomedical applications due to their intrinsic physicochemical properties. Their characteristics such as small size and high surface area give rise to unique features that allow their biomedical applications. The increasing demand for MNPs has attracted scientists to develop eco-friendly, straightforward, simple, and inexpensive processes for the enhanced production of MNPs. There are several methods used to synthesize metallic NPs. They are categorized into either bottom-up or top-down or biogenic green synthesis approaches. The list of efficient MNPs are gold, silver, Cu, Fe, *etc.*, and the bimetallic nanoparticles include Ag–Au, Fe–Co, Fe–Ni, Fe–Cu, Cu–Ni and Fe–Pt. Due to their unique electronic and optical profiles, chemical stability, and versatile surface chemistry, MNPs are ideal candidates for biomedical use. These properties, combined with ease of functionalization *via* surface charge, support their use in biosensing, imaging, and photothermal applications. For example, gold's chemical inertness allows for good biocompatibility *in vitro* and *in vivo*. Likewise, silver and its compounds are also used for medicinal purposes. For example, Ag-NPs have unique physicochemical properties such as high electrical conductivity, thermal conductivity, chemical stability, catalytic activity, antibacterial activities, and enhanced optical properties. Such properties have led to the use of Ag-NPs in antimicrobial and disinfectant applications. In addition, Ag-NPs are used in biomedical applications such as biosensing, photothermal therapy, and drug delivery. In conclusion, MNPs are used for various biomedical applications, and play multifunctional roles in therapeutics, diagnostics, imaging, and drug delivery. Various metallic and metal oxide nanoparticles are also used as scaffolds in tissue engineering and as biomaterials for medical applications.^217^

### 12.2 Future aspects

There are wide range of potential applications of metallic nanoparticles in the biomedical field. MNPs are mainly used in nano-diagnostics, which have the capability of enhancing the sensitivity and extending the present limits of molecular diagnostics/molecular imaging of various diseases. In other words, nanotechnology facilitates the development of high-performance biomarker recognition systems, biochips, and portable point-of-care devices. In addition, metallic nanomaterials show great potential for use as scaffolds in tissue engineering. Moreover, more studies and research are being conducted in therapeutic fields such as drug discovery, drug delivery, and gene/protein delivery. Other potential applications are medical diagnosis, treatment, and prevention of diseases. There is growing interest in the future medical applications of nanotechnology, leading to the emergence of a new field called nanomedicine.

#### 12.2.1 Utilization of biogenic or green-synthesized MNPs

In cancer therapies, traditional chemotherapeutic methods often face limitations due to poor bioavailability, rapid clearance, and severe side effects. Green synthesis emerges as an eco-friendly and cost-effective alternative for producing nanoparticles, leveraging natural sources like plant extracts to minimize environmental impact and enhance sustainability. This approach, operating under milder conditions, utilizes the versatility and scalability of green synthesis to create nanoparticles that exhibit improved biocompatibility and enhanced anticancer and antioxidant properties. The advancements of these biosynthesized MNPs in cancer nanomedicine, particularly their potential to induce apoptotic pathways and facilitate rapid penetration into cancer cells, offer promising therapeutic avenues for various cancer types including breast, prostate, skin, cervical, colorectal, lung, and liver cancers (M. Todaria *et al.*).^218^

#### 12.2.2 Functionalized MNPs in cancer therapy

The functionalization of MNPs with biomolecules like antibodies, DNA, or peptides substantially enhances their anticancer effects through mechanisms such as photothermal and photodynamic therapies, improved drug delivery, and increased specificity. These functionalized nanoparticles can effectively penetrate and accumulate in tumor tissue, leading to cytotoxic effects *via* ROS generation, apoptosis induction, cell cycle arrest, and DNA fragmentation. Consequently, functionalized metal nanoparticle-based therapy represents a novel strategy that can be synergistically combined with chemotherapy and radiotherapy to create more effective treatments with reduced side effects. Moreover, using biocompatible polymers, polysaccharides, proteins, dendrimers, and phase-stabilizers to achieve electrostatic or steric stabilization of GNP surfaces can provide more advancement in managing the tumor microenvironment and cancer management (K. Hariharan *et al.*).^219^

#### 12.2.3 Utilization of green-synthesized bi-metallic or conjugated metallic and magnetic nanoparticles

Different metal nanoparticles have their own advantages, for example, Au and Ag nanoparticles are good enough for the targeted drug delivery and photothermal therapy, whereas magnetic nanoparticles benefitted over for the imaging through MRI or hyperthermia *via* fluctuating the magnetic field. A conjugated or bimetallic nanoparticle can be a better candidate from targeted drug delivery to enhanced detection and imaging and efficient destruction of cancer cells and therapy of cancers (P. Srinoi *et al.*).^220^ For example, *Staphylococcus aureus*-mediated bimetallic silver–copper nanocomposites (Ag/Cu) with enhanced anticancer and antimicrobial properties are reported by M. A. Sayed *et al.*^221^ Two distinct approaches for preparing the bimetallic nanocomposites were adopted: one in which Ag and Cu ions were mixed before reduction (Ag/Cu-b), and another in which the ions were reduced separately and then mixed (Ag/Cu-a). The Ag/Cu-a nanocomposite demonstrated superior antimicrobial activity against a range of bacteria and fungi, and both types of nanocomposites exhibited promising anticancer activity against the hepatocellular carcinoma cell line (HepG-2) while showing low cytotoxicity against normal cells.

#### 12.2.4 Adequate approaches of combined photothermal therapies for the destruction and cure of the cancer

Heterobimetallic systems demonstrated the capacity to generate heat under either an alternating magnetic field or near-infrared laser light, validating their potential for magnetic hyperthermia and photothermal therapy. Nanoparticle theranostics are emerging as a promising solution for cancer treatments. Recently, surface-conjugated nanoparticles including lipid, metallic, and quantum dot nanoparticles, alongside platforms like plasmonic and magnetotheranostics, have shown positive preclinical results for solid tumor diagnosis and therapy. These innovative nanoparticle designs aim to precisely target tumors and selectively deliver imaging and therapeutic agents, minimizing harm to healthy tissues, improving hyperthermia and providing highly effective cancer treatment; however, further detailed research is required to overcome the existing limitations for full clinical utilization (V. P. Chavda *et al.*).^222^

## Conflicts of interest

There are no conflicts to declare.

## Abbreviations

NPs: Nanoparticles
MNPs: Metallic nanoparticles
Au-NPs: Gold nanoparticles
Ag-NPs: Silver nanoparticles
Cu-NPs: Copper nanoparticles
Fe-NPs: Iron nanoparticles
Se-NPs: Selenium nanoparticles
TiO_2_: Titanium dioxide
ZnO: Zinc oxide
FeO, Fe_2_O_3_: Iron oxide
Pt-NPs: Platinum nanoparticles
Se-NPs: Selenium nanoparticles
EBRT: External beam radiation therapy
IMRT: Intensity-modulated radiation therapy
GBMNPs: Green or biogenic metallic nanoparticles
DOX: Doxorubicin
HCC: Hepatocellular carcinoma
EPR: Enhanced permeability and retention
PDT: Photodynamic therapy
ROS: Reactive oxygen species
PTT: Photothermal therapy
CDT: Chemodynamic therapy
RT: Radiotherapy
Mg-NPs: Magnetic nanoparticles
PCE: Photothermal conversion efficiency
SPR: Surface plasmon resonance
LSPR: Localized surface plasmon resonances
SERS: Surface enhanced Raman scattering
CT: Computed tomography
XCT: X-ray computed tomography
XFS: X-ray fluorescence
MRI: Magnetic resonance imaging
US: Ultrasound

## Data Availability

No primary research results, software or code have been included, and no new data were generated or analyzed as part of this review.
